# Population differences in embryonic developmental timing in the lizard *Diploderma swinhonis*

**DOI:** 10.1371/journal.pone.0359078

**Published:** 2026-10-05

**Authors:** Jung-Ya Hsu, Chun-Chia Chou, Chen-Pan Liao, Ren-Chung Cheng, Wen-San Huang

**Affiliations:** 1 Department of Biology, National Museum of Natural Science, Taichung, Taiwan; 2 Department of Life Sciences, Chung Hsing University, Taichung, Taiwan; 3 Department of Life Science, National Taiwan Normal University, Taipei, Taiwan; 4 Department of Life Science, Tunghai University, Taichung, Taiwan; University of Missouri, UNITED STATES OF AMERICA

## Abstract

Geographic variation in reproductive timing can influence how embryonic development is partitioned between maternal egg retention and external incubation. We investigated population differences in embryonic developmental timing in the lizard *Diploderma swinhonis* by comparing eggs from Orchid Island and mainland Taiwan. Gravid females from mainland Taiwan and Orchid Island were collected and maintained under standardized laboratory conditions. Following oviposition, eggs within each clutch were sequentially dissected at 10-day intervals to minimize maternal effects and avoid pseudoreplication. Embryonic developmental stages were determined using a standardized developmental staging table, and developmental trajectories were compared between populations. Embryos from Orchid Island were deposited at significantly more advanced developmental stages, indicating that females retain eggs in the oviduct for a longer period prior to oviposition. In contrast, embryos from mainland Taiwan were laid at earlier developmental stages but subsequently exhibited faster post-oviposition developmental rates. Developmental synchrony also differed between populations, with embryos on Orchid Island showing lower synchrony and more gradual stage transitions among individuals. Despite these differences in developmental onset, rate, and synchrony, total incubation duration did not differ between populations, suggesting that embryonic development is partitioned differently between maternal retention and external incubation environments. Standardized maternal body size showed no detectable effect on developmental rate. These patterns may be associated with geographic differences in ecological conditions, including the presence of egg-eating snake predators on Orchid Island but not on mainland Taiwan. Such variation is consistent with facultative egg retention and highlights how reproductive timing can vary among populations within a species.

## Introduction

Reproductive timing is a central component of life-history variation because it determines the developmental state at which offspring enter external environments [1–3]. In oviparous reptiles, the embryonic stage at oviposition reflects the duration of intra-oviductal development prior to egg laying [4,5]. Most squamate reptiles deposit eggs during relatively early stages of embryogenesis, typically when major organ systems are established but differentiation remains incomplete [6,7]. Nevertheless, variation in embryonic stage at oviposition has been documented across taxa and, in some cases, among populations within species [8,9]. Such variation provides an opportunity to examine divergence in reproductive scheduling without invoking shifts between oviparity and viviparity.

Intra-oviductal egg retention has often been discussed in the context of the evolutionary transition from egg-laying to live-bearing reproduction [10–13]. However, quantitative differences in the duration of retention can occur entirely within oviparous systems and need not imply changes in reproductive mode [14]. Instead, embryonic stage at oviposition may vary along a continuum shaped by physiological constraints [15] and ecological context [16,17]. Maternal endocrine regulation [18], uterine oxygen availability [19], and abdominal capacity [12,15] may influence how long embryos can be retained prior to laying [4,5,12]. Female body size has been proposed as an important correlate because larger females may accommodate more advanced embryos or greater clutch mass before oviposition [20–22]. Consequently, accounting for maternal morphology is essential when evaluating population differences in developmental timing.

Geographic variation in reproductive traits is widespread in reptiles and often corresponds with environmental heterogeneity [17,23]. Climatic variables such as temperature and moisture can influence incubation environments and reproductive schedules [8,24]. Habitat structure and nest-site availability may also shape reproductive timing [25]. In addition, predator assemblages can differ among regions and alter the selective landscape experienced by eggs and nesting females [26]. Insular-mainland comparisons are particularly informative in this regard because island populations frequently experience modified predator communities, resource regimes, and demographic structure relative to mainland populations [27].

Diploderma is primarily distributed throughout Myanmar, China, Vietnam, Taiwan, and Japan [28]. *Diploderma swinhonis* is an oviparous agamid lizard endemic to Taiwan and belongs to the order Squamata and family Agamidae [28–31]. This species inhabits tropical forests, lowland woodlands, and urban environments, including school campuses and parks, and its breeding season generally extends from late March to late September [32]. In Taiwan, *D. swinhonis* occurs on both mainland Taiwan and Orchid Island, which is located approximately 63–72 km off the southeastern coast of mainland. Orchid Island populations experience intense predation pressure from the egg-eating snake *Oligodon formosanus*, which has been reported to consume a substantial proportion of small reptile clutches during the breeding season [28–31]. Mainland populations appear to support lower densities of this specialist predator. At the same time, large-scale climatic conditions are broadly similar between regions, reducing the likelihood that extreme thermal differences alone account for reproductive divergence. Despite extensive work on predator-prey interactions in this system, population-level differences in embryonic stage at oviposition have not been formally quantified.

Because embryonic stage can be assessed using standardized developmental tables [6], it provides a tractable and comparable metric for evaluating variation in reproductive timing. Here, we compare embryonic stage at oviposition between Orchid Island and mainland Taiwan populations of *D. swinhonis*. We test whether populations differ in developmental stage at laying while accounting for days after oviposition and maternal body size. Rather than attributing observed differences to a single ecological driver, our objective is to document the magnitude of interpopulation variation and provide a foundation for future studies investigating the ecological and physiological correlates of oviposition timing.

## Materials and methods

### Ethical approval

Dedication to ethics: All procedures were approved by the Institutional Animal Care and Use Committee of National Museum of Nature Science (NMNSIACUC2024−001), all methods were carried out in accordance with the relevant guidelines and regulations of NMNS-ICAUC, AVMA, and reported in accordance with ARRIVE guidelines in the study’s ethical application to NMNS-IACUC.

### Animal welfare guidelines for embryos and hatchlings

All experimental procedures were conducted in accordance with institutional animal care guidelines and approved animal use protocols. According to the Guide for the Care and Use of Laboratory Animals issued by the Ministry of Agriculture [33], euthanasia procedures for reptile embryos and hatchlings are determined according to developmental stage, with methods selected to minimize pain and distress. Early-stage embryos may be euthanized by freezing, whereas embryos at later developmental stages and hatchlings require appropriate chemical anesthesia or physical euthanasia.

### Euthanasia procedure

In the present study, euthanasia was performed using a physical method (decapitation) in accordance with the applicable animal welfare guidelines. No chemical anesthetic or analgesic was administered before euthanasia.

To clarify the experimental timeline, a schematic workflow is presented in Fig 1. Briefly, gravid *D. swinhonis* females were collected from both populations. Upon laboratory oviposition, eggs within each clutch were sequentially dissected at 10-day intervals to minimize maternal effects and pseudoreplication. Following embryonic staging, data were integrated into statistical models to evaluate inter-population developmental trajectories and stages at oviposition (Fig 1).

**Fig 1:**
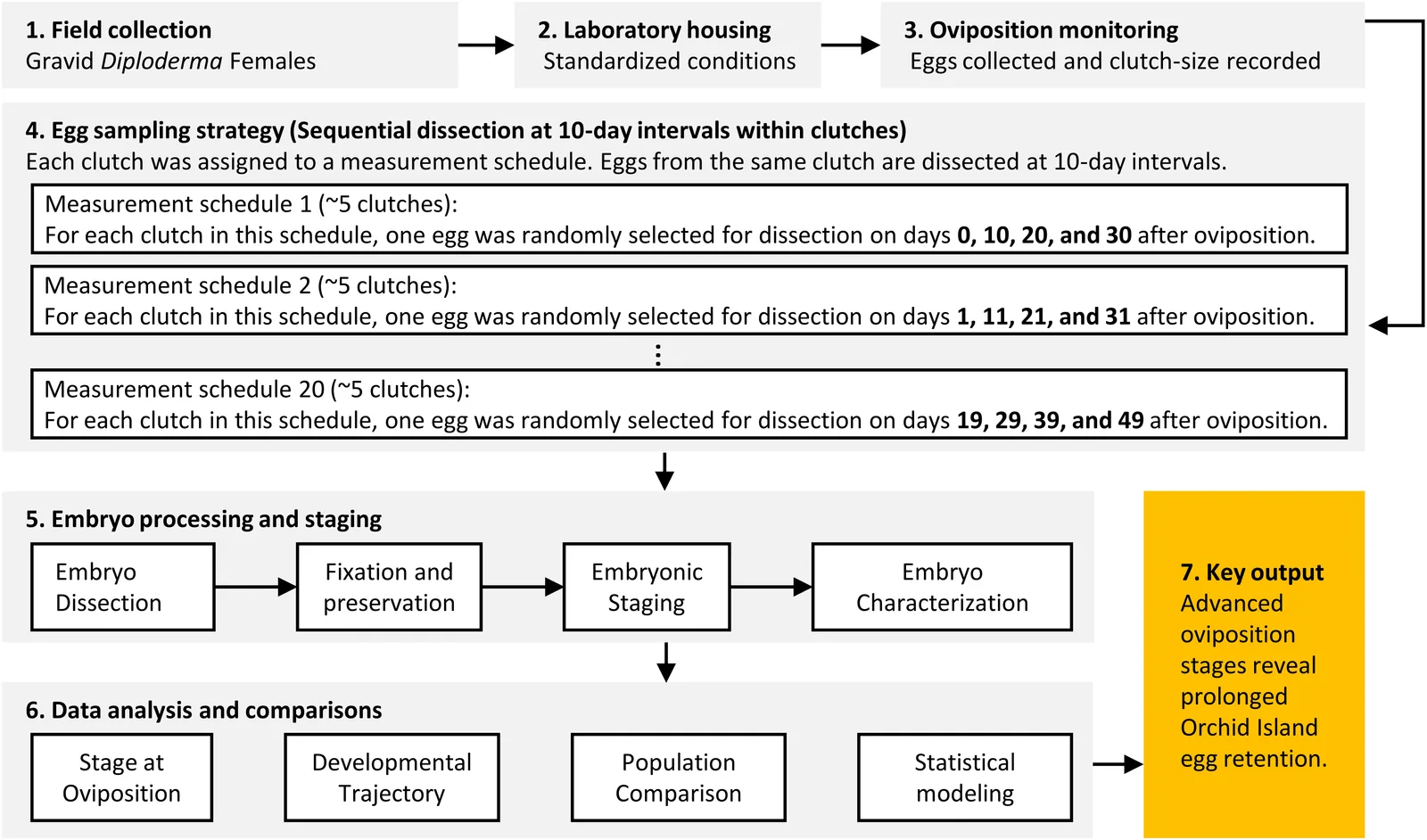
Schematic workflow of experimental design and data analysis. Sequential phases include: (1) field collection; (2) standardized housing; (3) oviposition monitoring; (4) within-clutch 10-day sequential sampling to minimize maternal effects; (5) embryo processing/staging; (6) statistical modeling; and (7) key biological output showing advanced oviposition stages and prolonged egg retention. Abbreviation: PO, post-oviposition.

### Animal collection and egg incubation

Gravid females that had already mated in the field were captured in the field from Orchid Island (22°03’27” N, 121°33’30” E) (N = 39) and mainland Taiwan (24°08’53” N, 120°33’06” E) (N = 36) and later kept in the laboratory for the 2024 breeding season. Each female was housed individually in a plastic container (34 × 17 × 24 cm) with 5 cm depth of soil substrate. We offered each female fresh water and mealworms (*Tenebrio molito*) ad libitum in the laboratory and released them back to the original capture sites after oviposition. Every egg deposited was labeled with the time of egg depositing and the date of dissection based on expected embryonic development. We maintained the room temperature at 29 ± 1°C, coupled with a 12/12h light/dark cycle throughout the experimental period [16,34]. Eggs were usually deposited in the daytime. We inspected these containers every day to confirm egg deposition. The collected eggs were placed in independent chambers covered with moist soil at a temperature of 29 ± 1°C [34].

### Embryo collection and developmental stages analyses

Gravid females were collected approximately 5–6 times, and females typically produced clutches of three to four eggs. Eggs were collected immediately after oviposition and assigned to predetermined dissection dates. The 10-day interval was selected because embryonic development in this species spans approximately 48–49 days, allowing major developmental transitions to be captured across incubation while ensuring that developmental sampling was distributed throughout the entire incubation period. Eggs were assigned to sampling dates at approximately 10-day intervals across multiple clutches. Because each female produced only 3–4 eggs, a single clutch could contribute eggs to only three or four sampling points. Sampling dates were therefore staggered among clutches; for example, eggs from one clutch were dissected on days 0, 10, 20, and 30, whereas eggs from another clutch were dissected on days 1, 11, 21, and 31. Additional clutches were similarly used to extend sampling across the incubation period. Each female contributed only one clutch, and no female contributed eggs to more than one clutch. This sequential within-clutch dissection design was used to avoid repeatedly dissecting multiple eggs from the same female on the same day, thereby minimizing maternal effects and reducing pseudoreplication in developmental time-series analyses.

Embryos were subsequently dissected and staged according to the developmental staging table of Dufaure and Hubert (1961) [6], originally developed for lacertid lizards and adapted here for staging embryonic development in *D. swinhonis*. During dissection, eggs were immersed in phosphate-buffered saline (PBS) and observed under a dissecting microscope (Leica MZ6). Embryos (N = 264) were fixed in 10% formalin solution for 2 hours (i.e., room temperature, RT). Egg dissections were performed under a stereomicroscope using fine dissection scissors and forceps. The eggshell was carefully opened, and surrounding extraembryonic tissues were gradually removed to expose the embryo for developmental staging. All dissections were conducted carefully to minimize mechanical damage to embryonic structures. After fixation, the embryos were stored in 75% ethanol at RT. To identify the embryo stage, we referred to the key morphological traits of different development stages of Squamata [6,35–37]. These traits included the pharyngeal clefts, brain, eyes, limbs, scale and pigmentation. We photographed embryos at different stages using a Canon EOS 850D camera equipped with either a Canon MP-E65 f2.8 1-5x Macro lens (stage 27–38) or Canon EF100mm f/2.8 Macro USM lens (stages 39–42)

### Statistical analyses

Embryonic developmental stage was analyzed using a Bayesian cumulative ordinal regression implemented in the R package brms [38] (version 2.23.0). This model was used to estimate the probability of each embryonic stage for eggs from different populations at a given post-oviposition time. Embryonic stage (*Y*) was treated as an ordered categorical response variable in a ordinal logistic regression. To link these discrete stages to the continuous process of embryonic development, we modeled latent developmental progress (*z*) as


$$\begin{array}{c} z=\text{exp}(r)(t-t_{\text{0}}), \end{array}$$

where *r* is the logarithm of the developmental rate, *t* is standardized post-oviposition time, and *t*_0_ is the developmental onset shift. The parameter *t*_0_ defines the estimated time point at which embryonic development begins within the maternal oviduct. This formulation allows developmental progress to increase linearly with time while constraining the developmental rate to be positive. The cumulative ordinal model estimated the probability that an egg was at or below embryonic stage *k* on day *t*:


$$\begin{array}{c} \text{P}(Y\leq k)={\text{logit}}^{-\text{1}}(d(\theta_{k}-z)), \end{array}$$

where *θ*_*k*_ is the threshold parameter separating stage *k* from later stages, and *d* is the discrimination parameter. Larger values of *d* indicate steeper transitions between stages and greater developmental synchrony. We modeled *r*, *t*_0_, and log(*d*) as functions of standardized maternal snout–vent length and lizard population using linear mixed models. Mother identity was included as a random intercept to account for non-independence among eggs from the same mother.

We report posterior medians and 95% equal-tailed credible intervals (CrI), probability of direction (PD) as an index of effect existence, and Bayes factors (BF), estimated using the Savage–Dickey density ratio, for the parameters of interest. Normal priors, (N (0, 1), were assigned to the regression coefficients of *r*, *t*_0_, and log(*d*). Student-(t) priors with 3 degrees of freedom, location 0, and scale 2 were assigned to the intercepts. Random-effect standard deviations were assigned half-Student-*t* priors with 3 degrees of freedom, location 0, and scale 2. Cutpoints in the ordinal model were assigned Student-*t* priors with 3 degrees of freedom, location 0, and scale 10.

## Results

We observed that embryos from mainland Taiwan were generally deposited at stages 27–28, whereas embryos from Orchid Island were deposited at the more advanced stage 31. Across incubation, embryos sampled at approximately 10-day intervals showed progressive developmental advancement, with embryos reaching approximately stages 33–34 after 10 days, 36 after 20 days, 39 after 30 days, and 41–42 after 40 days. Representative embryonic images from both populations were included to facilitate direct visual comparison of developmental stages between populations.

### Stage 27–30 (Fig 2)

In the stage 27, The optic cup became visible (Fig 2A). The first through third pharyngeal clefts were slightly discernible. The neural tube began to open dorsally from the metencephalon to the second pharyngeal cleft. The primordial region of hindlimbs slightly protruded. The forelimbs had not yet appeared. The tail bud without segments began to extend.

**Fig 2:**
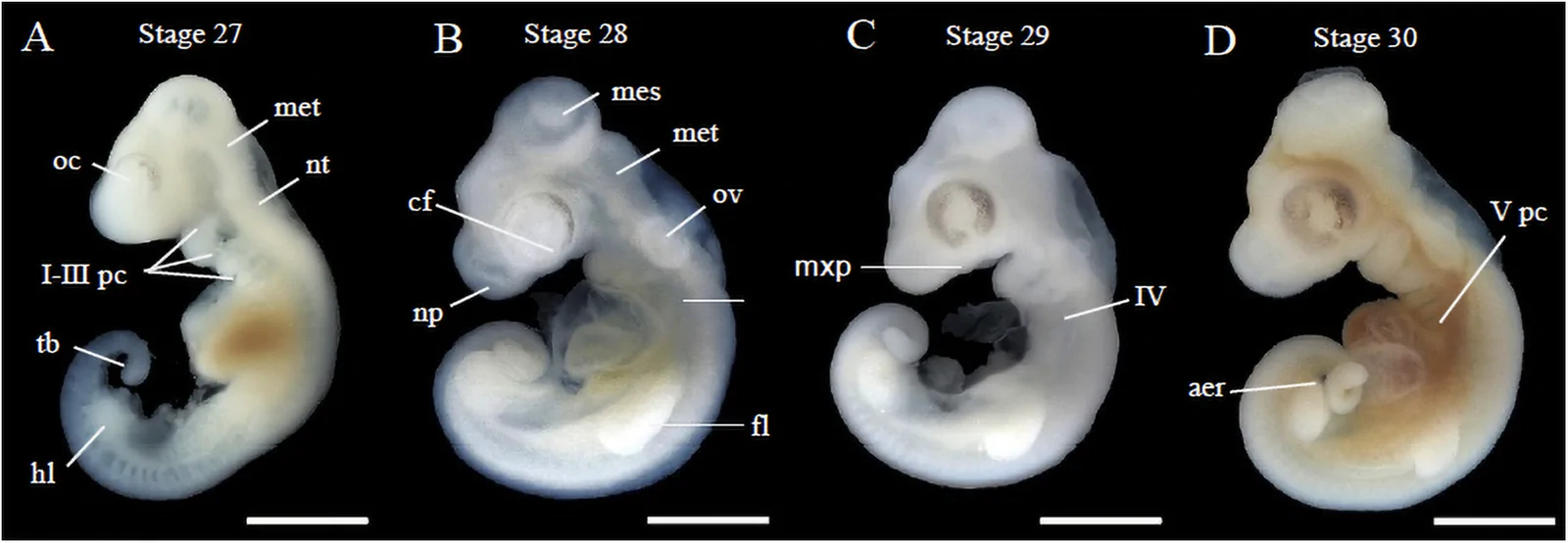
Post-ovipositional embryonic development (Stages 27-30) of *Diploderma swinhonis* from mainland Taiwan (TW), at stages 27-30. Scale bars = 1.0 mm. The stages are arranged in rows. An embryonic lateral view A-D. Abbreviations: aer, apical ectodermal ridge; cf, choroid fissure; fl, forelimb bud; hl, hindlimb bud; mes, mesencephalon; met, metencephalon; mxp, maxillary process; np, nasal pit; nt, neural tube; oc, optic capsule; ov, otic vesicle; tb, tail bud; I-III pc, I-III pharyngeal clefts; Ⅳ pc, IV pharyngeal cleft; V pc, V pharyngeal cleft.

We observed that the brain size increased with every region of the brain beginning to expand in the stage 28 (Fig 2B). The nasal pit located on the side of the nose was clear. The optic cup enlarged compared to the previous stage, and the choroid fissure started to open. The otic vesicle was visible and showed signs of calcium deposits. The first through third pharyngeal clefts were visible. The hindlimb buds appeared as round protuberances, while the forelimb buds were elongated protrusions. The tail bud remained unsegmented but started to curve.

In stage 29, the edges of the choroidal fissure were visible (Fig 2C). The dorsal region of the retina started to show pigmentation and became slightly visible. The first through fourth pharyngeal clefts could be clearly defined, with the fourth cleft faintly visible, and the maxillary bud was developing. The shapes of forelimb and hindlimb buds started to diverge, developing into distinct forms.

We observed the eye pigmentation began to develop in the stage 30 (Fig 2D). The nasal pits on the nasal prominences shifted toward the ventral surface. The otic vesicle underwent further calcification. The first through fifth pharyngeal clefts were clearly visible. The apical ectodermal ridges became well-defined on both the forelimb and hindlimb buds. During this stage, the tail bud elongated and started to coil.

### Stage 31–34 (Fig 3)

The telencephalon expanded and differentiated into distinct left and right hemispheres in the stage 31. Pigmentation increased across the entire ring-like iris. The second pharyngeal clefts nearly fully overlapped the third and fourth, making them difficult to distinguish. The maxillary processes aligned with the anterior margin of the iris. The forelimbs extended slightly beyond the hindlimbs, with more pronounced paddle-shaped digital plates. A subtle genital swelling was visible between the hindlimbs.

**Fig 3:**
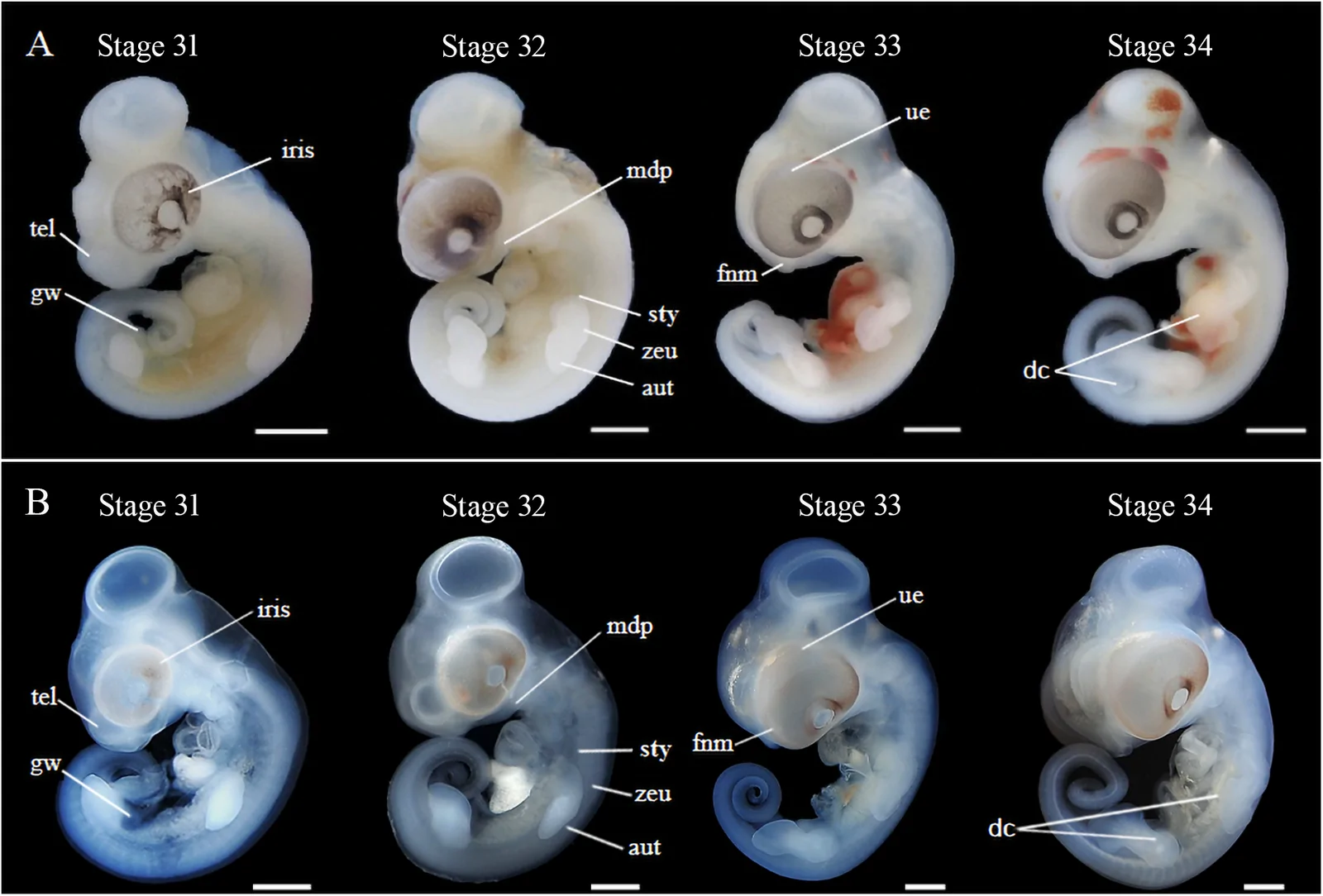
Post-ovipositional embryonic development (Stages 31-34) of *Diploderma swinhonis* from (A) mainland Taiwan (TW) and (B) Orchid Island (OI), at stages 31-34. Scale bars = 2.0 mm. An embryonic lateral view. Abbreviations: aut, autopod; dc, digital condensation; fnm, frontonasal mass; gw, genital swelling; iris; mdp, mandibular process; sty, stylopod; tel, telencephalon; ue, upper eyelid; zeu, zeugopod.

In the stage 32, the iris darkened as pigmentation increased. All pharyngeal clefts had fused and were no longer visible. The maxillary processes aligned with the anterior margin of the eye, while the mandibular processes were positioned posterior to the eye’s center. The stylopod, zeugopod, and autopod were distinct and well-differentiated. The width of the paddle-shaped digital plate expanded. The paired genital swellings became slightly more developed and prominent. Distinct flexures in both the forelimbs and hindlimbs allowed for clear differentiation of the stylopod, zeugopod, and autopod.

In the stage 33, the outline of the iris was clearly defined. The upper eyelid began to extend outward. The maxillary processes moved closer to the frontonasal prominences but had not yet made contact. Digital condensations were faintly visible.

In the stage 34, the outer edge of the iris was well-defined. The maxillary processes fused with the frontonasal processes, forming an external naris above the fusion point. The mandibular process extended anteriorly, reaching the level of the lens. Digital condensations of all five digits were clearly visible, and interdigital webbing began to form.

### Stage 35–38 (Fig 4)

In the stage 35, the upper eyelids were visible in a lateral view, while the lower eyelid emerged but did not yet overlap the iris. The maxillary and frontonasal processes fully fused. The mandibular process developed into the lower jaw, with its anterior tip meeting the tip of the upper jaw. Initial digital size differentiation occurred. Interdigital webbing showed deeper incisions at the digit tips, with a more pronounced separation between the fourth and fifth digits.

**Fig 4:**
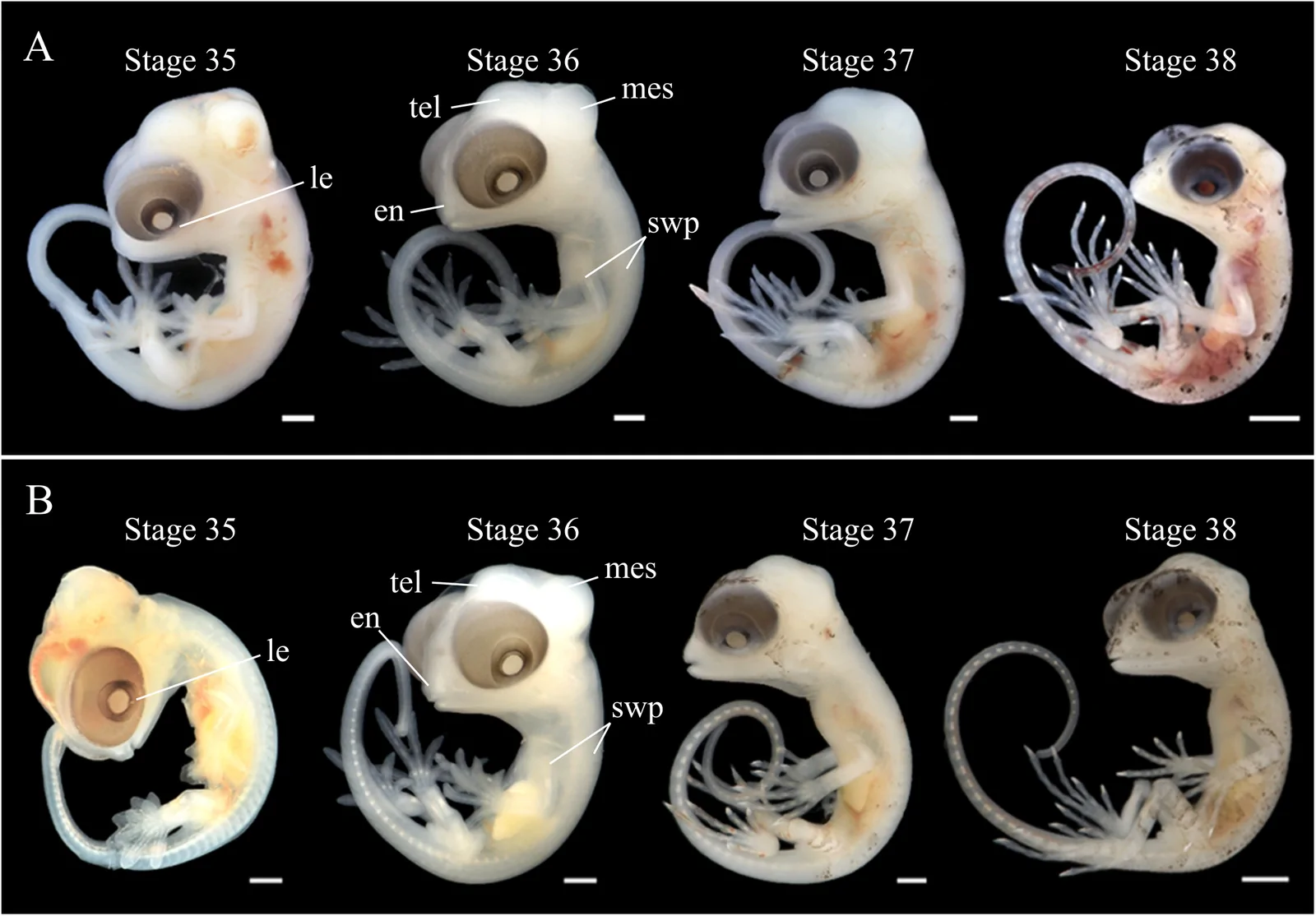
Post-ovipositional embryonic development (Stages 35-38) of *Diploderma swinhonis* from (A) mainland Taiwan (TW) and (B) Orchid Island (OI), at stages 35-38. Scale bars = 1.0 mm, at stages 38. Scale bars = 2.0 mm. The stages are arranged in rows. An embryonic lateral view. Abbreviations: en, external naris; le, lower eyelid; mes, mesencephalon; swp, scales without pigmentation; tel, telencephalon.

The telencephalon and mesencephalon were situated at the same horizontal level in the stage 36. The eyelid extended ventrally, partially covering the iris. The external naris remained a narrow slit. The lower jaw was equal in length to the upper jaw. Interdigital webbing was significantly reduced and the hindlimbs exhibited a notable elongation of the fourth digit compared to the others. Unpigmented scales began to emerge on the ventral surface of the trunk and the dorsal aspect of the limbs.

In the stage 37, the telencephalic and mesencephalic vesicles grew closer together. The upper eyelid had not yet overlapped the iris, while the lower eyelid extended beyond its lower boundary. The external naris presented a rounded configuration with a slight elevation. Interdigital webbing fully receded from all digits. The initial development of claws was evident, with a faint white coloration appearing at the distal end of the digits.

In the stage 38, the mesencephalon and telencephalon were closely positioned, causing a posterior slanting of the head. Both the upper and lower eyelids aligned with the level of the pupils. Tiny, sparsely pigmented spots appeared on the head and dorsal trunk. The limbs elongated with clearly defined segmentation. The claws were well-differentiated from the adjacent digits.

### Stage 39–42 (Fig 5)

In the stage 39, the mesencephalon and telencephalon exhibited a flattened morphology and came into direct contact. Claws on both forelimb and hindlimb reached full formation. Scale development increased on the dorsal surface of the head, neck, and trunk. The scale pattern became more defined, transitioning to a light brown hue along the body, accompanied by intensified pigmentation of the eyelids.

**Fig 5:**
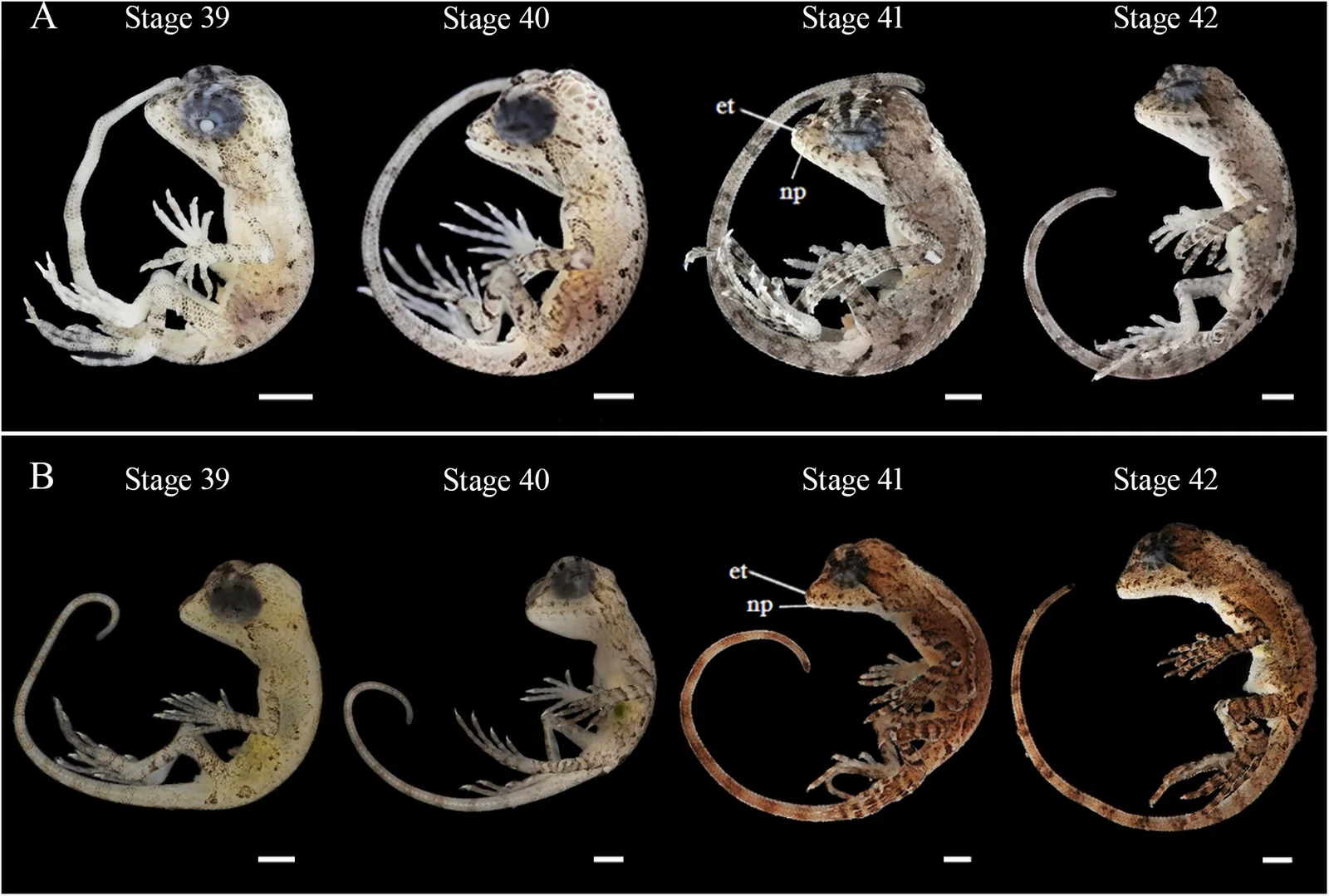
Post-ovipositional embryonic development (Stages 39-42) of *Diploderma swinhonis* from (A) mainland Taiwan (TW) and (B) Orchid Island (OI), at stages 39-42. Scale bars = 2.0 mm. The stages arranged in rows. An embryonic lateral view. Abbreviations: et, egg tooth; np, nasal pit.

In the stage 40, the mesencephalon and telencephalon were no longer visible. Both the upper and lower eyelids extended beyond the level of the pupil and were nearly closed. The pigmentation on fully developed scales became dark brown with distinct black and white spots and stripe patterns along the body.

In the stage 41, both the upper and lower eyelids were fully developed and completely closed. The scales had reached their final form. The pigmentation appeared slightly lighter compared to that of hatchlings. A single egg tooth protruded from the front tip of the upper jaw.

In the stage 42, the body took on a darker coloration, resembling the pigmentation of a newly hatched neonate. At this stage, the yolk was entirely consumed.

### Developmental rate, onset, and synchrony

The model revealed that embryos developed slightly more slowly on Orchid Island than on Taiwan. The estimated developmental rate was 0.68 stages per day on Orchid Island (95% CrI = 0.41–1.07) and 0.72 stages per day on Taiwan (95% CrI = 0.44–1.14). This corresponded to a 6.4% slower rate on Orchid Island (95% CrI = 2.7–10.6%), with moderate evidence for a site effect (PD = 99.9%, BF = 4.2; Fig 6). Maternal body size had no clear effect on developmental rate (*β* = 0.004, 95% CrI = −0.011 to 0.019, PD = 71.3%, BF = 0.009). Variation among mothers was small (SD = 0.017, 95% CrI = 0.001–0.051), suggesting that differences in developmental rate were mainly associated with population rather than individual mothers.

**Fig 6:**
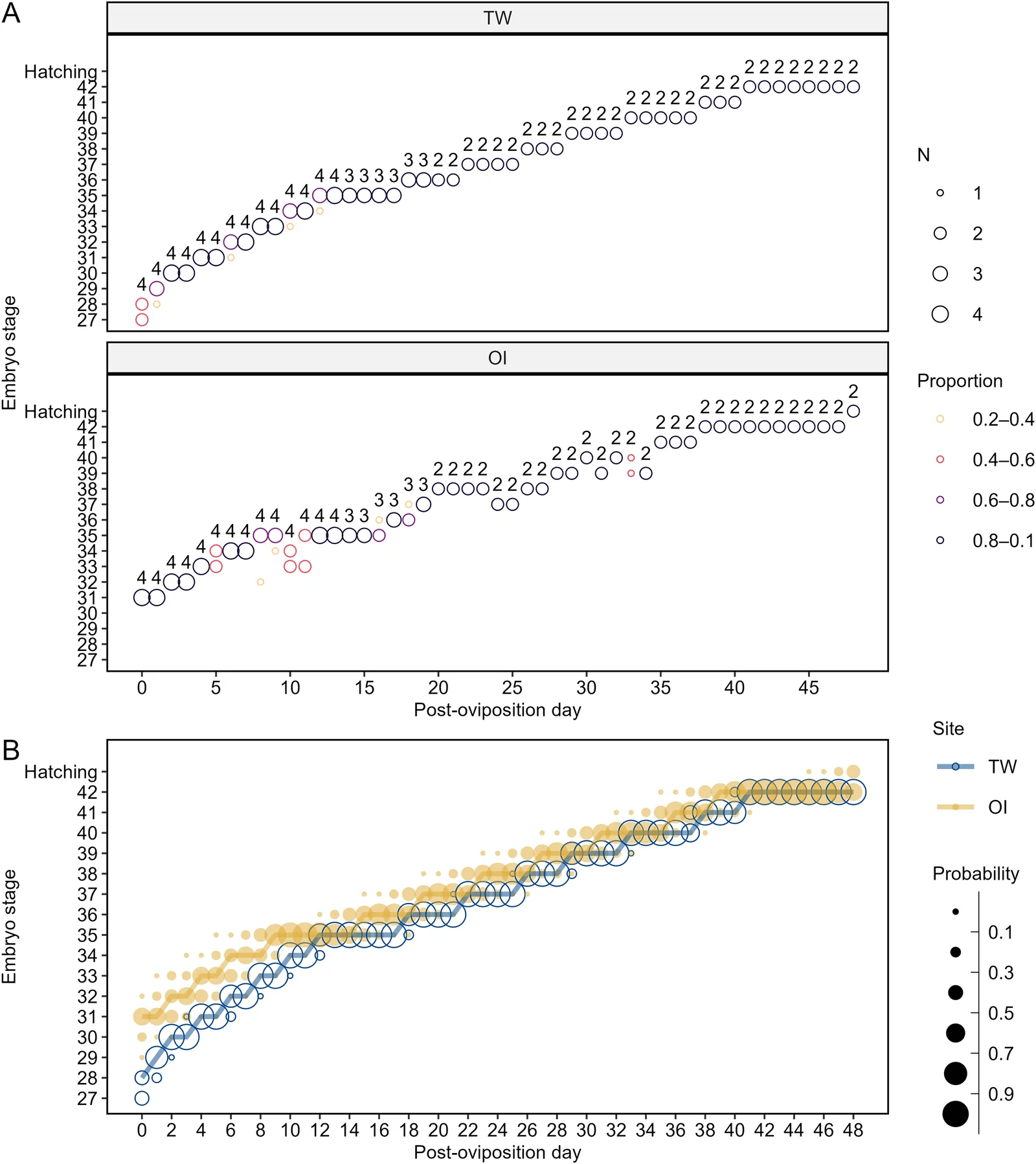
(A) Empirical sample distributions and stage proportions across sampling days: This panel presents the raw empirical distribution of sampled embryos. The upper grid represents mainland Taiwan (TW) and the lower grid represents Orchid Island (OI). Circle size (N) indicates the raw number of embryos dissected on that specific post-oviposition day (ranging from 2 to 4). The color coding of the circles represents the relative proportion of individuals at that specific developmental stage within that day’s sample. The numerical values above the symbols indicate the total replicate size (N) for clearer reference. (B) Bayesian model posterior predictions and trajectories: This panel displays the estimated continuous developmental trajectories derived from our Bayesian cumulative ordinal regression model. Blue hollow circles and lines represent the mainland Taiwan population (TW), while yellow filled circles and lines represent the Orchid Island population (OI). The solid trend lines represent the most probable embryonic stage predicted for each post-oviposition day. The size of the circles indicates the posterior probability (ranging from 0.1 to 0.9) of an embryo being at a given stage on a specific day, clearly demonstrating that Orchid Island embryos initiate development earlier, progress more slowly, and exhibit lower developmental synchrony.

Developmental onset also differed between sites. Embryos from Orchid Island were already more advanced at oviposition than embryos from Taiwan. This difference corresponded to approximately 3.5 additional days of development within the maternal oviduct (95% CrI = 2.7–4.5), with strong evidence for a site effect (PD = 100%, BF = 32106; Fig 6; S1 Table). Maternal body size had no clear effect on developmental onset (*β* = 0.002, 95% CrI = −0.006 to 0.009, PD = 67.8%, BF = 0.004). Variation among mothers was minimal (SD = 0.007, 95% CrI = 0.000–0.019).

Embryos from Orchid Island showed lower developmental synchrony than embryos from Taiwan. This was indicated by a lower discrimination parameter, log(*d*), on Orchid Island (*β* = −1.97, 95% CrI = −3.15 to −1.01), with strong evidence for a site effect (PD = 100%, BF = 2166354; Fig 6). Because lower values of log(*d*) indicate more gradual transitions among stages, this result suggests that embryonic stages were more variable on Orchid Island. Maternal body size had no clear effect on developmental synchrony (*β* = −0.21, 95% CrI = −0.64 to 0.27, PD = 82.9%, BF = 0.37). Variation among mothers was moderate (SD = 0.51, 95% CrI = 0.05–1.14).

Taken together, embryos on Orchid Island initiated development slightly earlier, developed more slowly (Fig 6; S1 Table), and exhibited lower developmental synchrony (Fig 6; S1 Table) than those on Taiwan. Consequently, embryos on Orchid Island remained at more advanced stages until approximately 45 days after oviposition (Fig 6; S1 Table). The estimated time to reach hatching was similar between sites. Embryos on Orchid Island reached stage 43 at a median of 48.4 days after oviposition (95% CrI = 46.9 to 51.1), compared with 49.5 days after oviposition for embryos on Taiwan (95% CrI = 48.1 to 51.9).

## Discussion

The present study demonstrates clear population-level differences in embryonic developmental timing in *D. swinhonis*. Eggs from Orchid Island were deposited at significantly more advanced developmental stages than those from mainland Taiwan, indicating prolonged intra-oviductal egg retention in the Orchid Island population. Because embryos from both populations were subsequently maintained under identical laboratory conditions, the observed differences are unlikely to result solely from post-oviposition environmental effects. Instead, these findings suggest divergence in reproductive timing and maternal regulation of embryonic development between geographically isolated populations.

Developmental staging further revealed that embryos from both populations followed broadly similar morphological trajectories during incubation, indicating that the primary difference lies in the timing of oviposition rather than major divergence in developmental patterning itself. Thus, the populations appear to differ mainly in developmental timing (heterochrony) rather than developmental sequence [39]. Such heterochronic shifts may represent an adaptive mechanism that allows populations to respond to local ecological pressures while maintaining conserved developmental pathways.

Developmental synchrony significantly differed between populations, with Orchid Island embryos exhibiting greater individual variation post-oviposition. It suggests that prolonged maternal egg retention may fundamentally alter intrinsic developmental dynamics. From an evolutionary perspective, this lower synchrony may serve as an adaptive bet-hedging strategy under intense insular predation pressure. Reptilian embryos at late stages exhibit peak metabolic rates, releasing significant volatile organic compounds that create a concentrated “olfactory target” for specialized egg-eating predators, such as *O formosanus* on Orchid Island [31,40]. Conversely, asynchronous development temporally staggers these individual metabolic peaks, potentially diluting the overall chemical signal to evade detection. Furthermore, such asynchrony naturally extends the hatching window among siblings, reducing the risk of cohort-wide mortality or synchronized predator swarming during emergence. Therefore, prolonged retention may function as a multi-layered reproductive strategy shaping subsequent embryogenesis to maximize offspring survival in high-risk ecosystems.

Standardized maternal body size showed no detectable effect on developmental rate, suggesting that variation in embryonic timing is unlikely to be explained simply by female morphology. Instead, the observed population differences are more consistent with ecological influences on reproductive timing. Such patterns are consistent with facultative egg retention [41,42], in which females adjust the duration of intra-oviductal embryo retention in response to local ecological conditions. Facultative egg retention has been documented in several squamate reptiles and is frequently interpreted as an intermediate reproductive strategy along the evolutionary continuum between oviparity and viviparity [14,43]. The contrasting developmental patterns observed between Orchid Island and mainland Taiwan populations therefore suggest that geographic variation in ecological conditions may shape maternal control over embryonic development and reproductive timing.

The distinctive embryonic developmental pattern observed in the Orchid Island population may also be associated with the unique ecological context of this island, particularly the exceptionally high predation pressure imposed by the egg-eating snake *O. formosanus*. Previous studies have demonstrated that Orchid Island supports unusually high densities of this predator due to the seasonal availability of sea turtle nests, which subsequently increases predation pressure on reptile eggs, including those of lizards. Strong egg predation pressure on Orchid Island has already been linked to several remarkable evolutionary responses in insular lizards. For example, Orchid Island populations of the long-tailed skink (*Eutropis longicaudata*) exhibit post-ovipositional maternal care in the form of nest guarding behavior, a trait absent in mainland populations [30]. Experimental studies further demonstrated that this maternal care behavior evolved specifically as a defense against egg predation by *O. formosanus*. In addition, Orchid Island skinks exhibit significantly greater bite force than mainland populations, likely as an adaptive response associated with parental defense and predator deterrence.

Environmental temperature is known to influence developmental rates and reproductive traits in reptiles. However, mainland Taiwan and Orchid Island occur at similar latitudes and share broadly comparable climatic conditions and habitats, reducing the likelihood that large-scale environmental temperature differences alone explain the observed developmental divergence. Historical climatic records support this interpretation. According to the 1991–2020 climate normals provided by the Central Weather Administration (CWA) [44], Taiwan (Taichung) and Orchid Island have broadly similar annual mean temperatures (23.3°C and 23.0°C, respectively), whereas Orchid Island receives substantially greater annual precipitation than Taiwan (Taichung) (3,237.5 vs. 1,773.0 mm). Although these precipitation differences do not directly predict the pattern of prolonged egg retention observed in our study, rainfall may influence reproductive timing through its effects on female oviposition decisions and local nesting conditions. In *Anolis aeneus*, females can retain shelled oviducal eggs during drought, whereas rainfall facilitates oviposition when soil moisture is sufficient [45]. Similarly, García-Collazo et al. [46] found that egg retention was negatively related to precipitation, whereas environmental temperature had no significant effect. Thus, although the higher annual precipitation on Orchid Island does not readily explain its prolonged egg retention, seasonal or local variation in rainfall and soil moisture may influence maternal reproductive timing and the developmental stage at oviposition. The subsequent embryonic developmental differences observed after oviposition are therefore more likely to reflect population-specific developmental dynamics than broad-scale climatic differences.

When integrated into this ecological context, the advanced embryonic stages observed at oviposition in Orchid Island *D. swinhonis* may represent another reproductive response to intense egg predation pressure. Prolonged intra-oviductal egg retention could reduce the duration of vulnerable external incubation, thereby shortening the period during which eggs remain exposed to predators. Although the present study does not directly test the adaptive fitness consequences of egg retention, the observed developmental divergence is consistent with the hypothesis that predator-driven selection on Orchid Island has shaped multiple reproductive and developmental traits in reptiles inhabiting this insular ecosystem. More broadly, these findings support the idea that reproductive timing, parental investment, and embryo retention can evolve rapidly under strong ecological pressures. The Orchid Island population therefore provides a valuable natural system for understanding how localized predation pressure may influence the evolution of reproductive strategies and embryonic developmental timing in oviparous reptiles.

## Supporting information

S1 Table Posterior estimates of developmental-stage transition times (50% threshold-crossing probability) for the Taiwan and Orchid Island populations. Values represent posterior medians and 95% credible intervals for the estimated day at which embryos cross each developmental-stage threshold (day 0  =  oviposition). Negative values indicate that the estimated crossing occurs before oviposition.
(DOCX)
