# Supplementary material for: Population differences in embryonic developmental timing in the lizard Diploderma swinhonis
Source: PLoS One. 2026 Oct 5;21(10):e0359078. doi: 10.1371/journal.pone.0359078 (PMC13637867; doi:10.1371/journal.pone.0359078)
Supplement: Supplementary file 1 [file pone.0359078.s001.docx]

| Stage transition | Taiwan (days) | Orchid Island (days) |
| --- | --- | --- |
| 27 to 28 | −0.0 [−0.7, 0.4] | −4.6 [−5.6, −3.7] |
| 28 to 29 | 0.8 [0.3, 1.2] | −3.7 [−4.6, −2.8] |
| 29 to 30 | 1.6 [1.1, 2.0] | −2.9 [−3.8, −2.0] |
| 30 to 31 | 3.5 [3.0, 4.0] | −0.8 [−1.6, −0.1] |
| 31 to 32 | 5.8 [5.3, 6.2] | 1.6 [0.9, 2.3] |
| 32 to 33 | 7.6 [7.2, 8.0] | 3.6 [3.0, 4.2] |
| 33 to 34 | 9.7 [9.3, 10.1] | 5.9 [5.3, 6.5] |
| 34 to 35 | 11.9 [11.5, 12.2] | 8.1 [7.6, 8.7] |
| 35 to 36 | 17.9 [17.4, 18.4] | 14.6 [14.0, 15.3] |
| 36 to 37 | 21.4 [20.9, 22.0] | 18.4 [17.7, 19.1] |
| 37 to 38 | 25.2 [24.6, 25.8] | 22.4 [21.7, 23.2] |
| 38 to 39 | 28.9 [28.2, 29.7] | 26.3 [25.5, 27.5] |
| 39 to 40 | 32.7 [32.1, 33.5] | 30.4 [29.5, 31.6] |
| 40 to 41 | 37.1 [36.1, 37.8] | 35.1 [34.1, 36.1] |
| 41 to 42 | 40.1 [39.1, 40.9] | 38.3 [37.4, 39.4] |
| 42 to hatching | 49.5 [48.1, 51.8] | 48.4 [46.8, 51.1] |
