# Supplementary material for: Population differences in embryonic developmental timing in the lizard Diploderma swinhonis
Source: PLoS One. 2026 Oct 5;21(10):e0359078. doi: 10.1371/journal.pone.0359078 (PMC13637867; doi:10.1371/journal.pone.0359078)
Supplement: Supplementary file 4 [file pone.0359078.s005.docx]

**Response to Reviewers**

Manuscript ID: ONE-D-26-13257
Title: *Population differences in embryonic developmental timing in the lizard Diploderma swinhonis*

Dear Academic Editor and Reviewers,

We sincerely thank the Academic Editor and the reviewers for their constructive comments and valuable suggestions. We have carefully considered all of the comments and revised the manuscript accordingly. All revisions are indicated in the tracked-changes version of the manuscript, and our detailed responses to each comment are provided below.

**Responses to Reviewer #3**

**Comment 1:**

The study is interesting, and the results are significant. However, I find it hard to believe that the fact that it rains almost twice as much in Orchid as in Taiwan isn't just another variable affecting egg retention. And it’s very clear that it doesn’t directly affect the eggs, since they’re incubated under laboratory conditions, but it is affecting the females. I think the authors should make it sufficiently clear that it is the females who would be affected by the rains and the abundance of predators, which would cause them to retain their eggs. These Orchid eggs accelerate their embryonic development so that they hatch in the same number of days as they do in Taiwan.

**Response to reviewer:**

Thank you for this important comment. We agree that rainfall may indirectly influence egg retention and oviposition decisions through its effects on females and local nesting conditions. We have therefore added a brief discussion of previous studies showing that rainfall and soil moisture can influence egg retention and oviposition timing in lizards [45,46]. We now acknowledge that seasonal or local variation in rainfall may contribute to maternal reproductive timing and the developmental stage at oviposition.

**(Lines 417–436)**

**Discussion**

“Environmental temperature is known to influence developmental rates and reproductive traits in reptiles. However, mainland Taiwan and Orchid Island occur at similar latitudes and share broadly comparable climatic conditions and habitats, reducing the likelihood that large-scale environmental temperature differences alone explain the observed developmental divergence. Historical climatic records support this interpretation. According to the 1991–2020 climate normals provided by the Central Weather Administration (CWA)[44], Taiwan (Taichung) and Orchid Island have broadly similar annual mean temperatures (23.3°C and 23.0°C, respectively), whereas Orchid Island receives substantially greater annual precipitation than Taiwan (Taichung) (3,237.5 vs. 1,773.0 mm). Although these precipitation differences do not directly predict the pattern of prolonged egg retention observed in our study, rainfall may influence reproductive timing through its effects on female oviposition decisions and local nesting conditions. In *Anolis aeneus*, females can retain shelled oviducal eggs during drought, whereas rainfall facilitates oviposition when soil moisture is sufficient [45]. Similarly, García-Collazo et al. [46] found that egg retention was negatively related to precipitation, whereas environmental temperature had no significant effect. Thus, although the higher annual precipitation on Orchid Island does not readily explain its prolonged egg retention, seasonal or local variation in rainfall and soil moisture may influence maternal reproductive timing and the developmental stage at oviposition. The subsequent embryonic developmental differences observed after oviposition are therefore more likely to reflect population-specific developmental dynamics than broad-scale climatic differences.”

**Comment 2:**

I recommend publishing the study, and I would even suggest that the authors review the final table they present to see if it might be better to present it as a line or scatter plot (with the respective standard deviations).

**Response to reviewer:**

Thank you for this helpful suggestion. We carefully considered whether Fig 6 would be better presented as a line or scatter plot with standard deviations. However, we believe that the format is more appropriate for our study because Fig 6 is intended to provide the developmental data and statistical comparisons across sampling stages and populations, rather than to emphasize a continuous relationship or trend. We therefore retained Fig 6 in its current format to allow readers to directly compare the developmental stages and associated variation among populations.
