# Supplementary material for: Population differences in embryonic developmental timing in the lizard Diploderma swinhonis
Source: PLoS One. 2026 Oct 5;21(10):e0359078. doi: 10.1371/journal.pone.0359078 (PMC13637867; doi:10.1371/journal.pone.0359078)
Supplement: Supplementary file 6 [file pone.0359078.s004.docx]

**Response to Reviewers**

Manuscript ID: ONE-D-26-13257
Title: *Population differences in embryonic developmental timing in the lizard Diploderma swinhonis*

Dear Academic Editor and Reviewers,

We sincerely thank the Academic Editor and reviewers for their constructive comments and valuable suggestions. We have carefully revised the manuscript according to all comments received.

All changes in the revised manuscript are highlighted in the tracked-changes version.

Detailed responses to each comment are provided below.

**Responses to the Academic Editor**

**Comment 1:**

While I understand that this version was greatly improved, Reviewer 3 suggests that authors should consider the differences in climatic conditions between sites, if they exist. This is briefly mentioned in the introduction and I agree with the reviewer´s assessment that this should not be overlooked.

(The Discussion, Line 421-435)

**Response to Academic Editor:**

Thank you for highlighting this important point. We agree that climatic differences between the two study sites should not be overlooked when interpreting geographic variation in embryonic developmental timing. We have therefore added historical climatic information for Taiwan (Taichung) and Orchid Island based on the 1991-2020 climate normal provided by the Central Weather Administration (CWA), Taiwan. The two regions have broadly similar annual mean temperatures (23.3°C in Taichung and 23.0°C on Orchid Island), whereas Orchid Island receives substantially greater annual precipitation than Taichung (3,237.5 vs. 1,773.0 mm).

We have incorporated these climatic differences into the Discussion (Lines 421–435) and considered their potential relevance to maternal egg retention and embryonic development before oviposition. We also clarify that, because eggs from both populations were subsequently incubated under identical laboratory conditions, regional climatic differences cannot directly explain differences in the environmental conditions experienced during post-oviposition incubation. Thus, local climate is considered a potential ecological factor that may influence embryonic developmental state before oviposition, rather than a direct explanation for the developmental trajectories observed under standardized laboratory conditions.

**Comment 2:**

Lines 112-116: Please indicate the exact method of anesthesia utilized during euthanasia, indicating which anesthetic was used, precedence (supplier, country) and the dose used.

**Response to Academic Editor:**

Thank you for pointing this out. We have clarified the euthanasia procedure in the revised Methods section. No chemical anesthetic or analgesic was used in this study. Euthanasia was performed using a physical method in accordance with the animal care guidelines and the approved institutional animal use protocol. Therefore, information regarding anesthetic type, supplier, country of origin, and dosage is not applicable. The Methods section has been revised to explicitly state that euthanasia was performed without chemical anesthesia or analgesia.

**(Lines 104–115)**

**Revised Methods**

**“****Animal Welfare Guidelines for Embryos and Hatchlings**
All experimental procedures were conducted in accordance with institutional animal care guidelines and approved animal use protocols. According to the Guide for the Care and Use of Laboratory Animals issued by the Ministry of Agriculture [34], euthanasia procedures for reptile embryos and hatchlings are determined according to developmental stage, with methods selected to minimize pain and distress. Early-stage embryos may be euthanized by freezing, whereas embryos at later developmental stages and hatchlings require appropriate chemical anesthesia or physical euthanasia.

**Euthanasia Procedure**
In the present study, euthanasia was performed using a physical method (decapitation) in accordance with the applicable animal welfare guidelines. No chemical anesthetic or analgesic was administered before euthanasia.”

**Comment 3:**

Lines 150-151: The authors mention that the eggs were dissected at sampling day (day 0), then again at days 10, 20, 30 and 40. This accounts for a total of 5 eggs, although the authors inform that clutches had 3-4 eggs (line 145). Additionally, in the new Figure 1, the authors indicate that eggs were dissected only until day 30. Please correct accordingly.

**Response to Academic Editor:**

Thank you for pointing this out. We apologize that the sampling design was not sufficiently clear in the previous version. The 10-day interval refers to the overall sampling schedule across multiple clutches, rather than to five eggs from the same female. Each gravid female produced only 3-4 eggs, and therefore a single clutch could contribute eggs to only three or four sampling points. For example, eggs from one clutch could be dissected on days 0, 10, 20, and 30, while eggs from another clutch were assigned to days 1, 11, 21, and 31. Similarly, additional clutches were used to progressively cover later sampling points. Thus, the day 40 samples were obtained from different clutches rather than from the same female's clutch.

We have revised the Methods section and Figure 1 to clarify that the 10-day sampling interval was achieved across multiple clutches, with sampling days staggered among clutches. This design allowed us to characterize embryonic development across the full incubation period while respecting the natural clutch size of *D. swinhonis*.

**(Lines 147–154)**

**Methods**

“Eggs were assigned to sampling dates at approximately 10-day intervals across multiple clutches. Because each female produced only 3-4 eggs, a single clutch could contribute eggs to only three or four sampling points. Sampling dates were therefore staggered among clutches; for example, eggs from one clutch were dissected on days 0, 10, 20, and 30, whereas eggs from another clutch were dissected on days 1, 11, 21, and 31. Additional clutches were similarly used to extend sampling across the incubation period. Each female contributed only one clutch, and no female contributed eggs to more than one clutch.”

**Figure 1**

Thank you for pointing this out. We agree that the original wording in Figure 1 could be misleading, as it may imply that eggs from the same clutch were dissected at all sampling intervals. To clarify the sampling design, we have revised the description in Figure 1 from **“Each clutch contains 3-4 eggs; eggs from the same clutch are dissected at 10-day intervals”** to **“Each clutch was assigned to a measurement schedule. Eggs from the same clutch are dissected at 10-day intervals.”** This revised wording more accurately reflects that the sampling schedule was distributed across multiple clutches, with sampling dates staggered to obtain developmental data at approximately 10-day intervals.

**Responses to Reviewer #3**

**Comment 1:**

The study is interesting, and the information it provides is presented in a clear and concise manner. However, I believe that the study should not overlook the climatic conditions under which these females live (both on Orchid Island and in Taiwan). I think it is necessary to add to the manuscript and briefly discuss (using historical records) the climatic conditions that could be affecting both egg retention and environmental incubation. Perhaps rainfall or temperature can better explain the slower development of the embryos on Orchid Island during (external) incubation.

**Response to reviewer:**

Thank you for this important suggestion. We agree that the climatic conditions experienced by the two populations should be considered when interpreting geographic variation in embryonic developmental timing. We have therefore added historical climatic information for Taiwan (Taichung) and Orchid Island based on the 1991-2020 climate normal provided by the Central Weather Administration (CWA), Taiwan. The two regions have broadly similar annual mean temperatures (23.3°C in Taiwan (Taichung) and 23.0°C on Orchid Island), whereas Orchid Island receives substantially more annual precipitation (3,237.5 mm) than Taichung (1,773.0 mm).

We have added a brief discussion of these climatic differences and their potential relevance to maternal egg retention and embryonic development **(Lines 421-435).** Importantly, because eggs from both populations were subsequently incubated under standardized laboratory conditions, regional climatic differences are unlikely to directly explain the differences in post-oviposition developmental trajectories. Instead, local climatic conditions may have influenced embryonic development before oviposition, including the duration of intra-oviductal egg retention. We have therefore revised the Discussion to acknowledge climate as a potential ecological factor while avoiding causal interpretation beyond the evidence provided by our experimental design.

**(Lines 421–435)**

**Discussion**

“Historical climatic conditions may also contribute to geographic variation in embryonic developmental timing. According to the 1991–2020 climate normal provided by the Central Weather Administration (CWA) [44], Taichung and Orchid Island have broadly similar annual mean temperatures (23.3°C and 23.0°C, respectively), whereas Orchid Island receives substantially greater annual precipitation than Taichung (3,237.5 vs. 1,773.0 mm). These climatic differences may influence maternal reproductive physiology and embryonic development before oviposition, potentially affecting the duration of intra-oviductal egg retention. However, because eggs from both populations were incubated under identical laboratory conditions after oviposition, regional climatic differences cannot directly account for differences in the environmental conditions experienced during post-oviposition incubation. Thus, local climate may represent an ecological factor contributing to developmental differences established before oviposition, whereas the subsequent developmental trajectories observed under standardized conditions are more likely to reflect population-specific developmental dynamics.”
