# Supplementary material for: Population differences in embryonic developmental timing in the lizard Diploderma swinhonis
Source: PLoS One. 2026 Oct 5;21(10):e0359078. doi: 10.1371/journal.pone.0359078 (PMC13637867; doi:10.1371/journal.pone.0359078)
Supplement: Peer review history

# Peer review: Decision Letter 0

**DOI:** 10.1371/journal.pone.0359078.r001

**Authors:** Renato Filogonio

12 May 2026

Dear Dr. Huang,

Thank you for submitting your manuscript to PLOS ONE. After careful consideration, we feel that it has merit but does not fully meet PLOS ONE’s publication criteria as it currently stands. Therefore, we invite you to submit a revised version of the manuscript that addresses the points raised during the review process.

==============================

**ACADEMIC EDITOR:**

Both reviewers had concerns about the methodology used, which should be carefully addressed in the next version of the manuscript. Please find bellow their comments and suggestions.

==============================

Please submit your revised manuscript by Jun 26 2026 11:59PM. If you will need more time than this to complete your revisions, please reply to this message or contact the journal office at plosone@plos.org. When you're ready to submit your revision, log on to https://www.editorialmanager.com/pone/ and select the 'Submissions Needing Revision' folder to locate your manuscript file.

Please include the following items when submitting your revised manuscript:

- A letter that responds to each point raised by the academic editor and reviewer(s). You should upload this letter as a separate file labeled 'Response to Reviewers'.
- A marked-up copy of your manuscript that highlights changes made to the original version. You should upload this as a separate file labeled 'Revised Manuscript with Track Changes'.
- An unmarked version of your revised paper without tracked changes. You should upload this as a separate file labeled 'Manuscript'.

If you would like to make changes to your financial disclosure, please include your updated statement in your cover letter. Guidelines for resubmitting your figure files are available below the reviewer comments at the end of this letter.

If applicable, we recommend that you deposit your laboratory protocols in protocols.io to enhance the reproducibility of your results. Protocols.io assigns your protocol its own identifier (DOI) so that it can be cited independently in the future. For instructions see: https://journals.plos.org/plosone/s/submission-guidelines#loc-laboratory-protocols. Additionally, PLOS ONE offers an option for publishing peer-reviewed Lab Protocol articles, which describe protocols hosted on protocols.io. Read more information on sharing protocols at https://plos.org/protocols?utm_medium=editorial-email&utm_source=authorletters&utm_campaign=protocols.

As the corresponding author, your ORCID iD is verified in the submission system and will appear in the published article. PLOS supports the use of ORCID, and we encourage all coauthors to register for an ORCID iD and use it as well. Please encourage your coauthors to verify their ORCID iD within the submission system before final acceptance, as unverified ORCID iDs will not appear in the published article. *Only*  the individual author can complete the verification step; PLOS staff *cannot*  verify ORCID iDs on behalf of authors.

We look forward to receiving your revised manuscript.

Kind regards,

Renato Filogonio

Academic Editor

PLOS One

Journal Requirements:

When submitting your revision, we need you to address these additional requirements.

1. Please ensure that your manuscript meets PLOS ONE's style requirements, including those for file naming. The PLOS ONE style templates can be found at

https://journals.plos.org/plosone/s/file?id=wjVg/PLOSOne_formatting_sample_main_body.pdf and

https://journals.plos.org/plosone/s/file?id=ba62/PLOSOne_formatting_sample_title_authors_affiliations.pdf

2. To comply with PLOS One submissions requirements, in your Methods section, please provide additional information regarding the experiments involving animals and ensure you have included details on (1) methods of sacrifice, (2) methods of anesthesia and/or analgesia, and (3) efforts to alleviate suffering.

3. Thank you for stating the following financial disclosure:

"MOST 113-2621-B-178-001- MY3"

Please state what role the funders took in the study.  If the funders had no role, please state: "The funders had no role in study design, data collection and analysis, decision to publish, or preparation of the manuscript."

If this statement is not correct you must amend it as needed.

Please include this amended Role of Funder statement in your cover letter; we will change the online submission form on your behalf.

4. Thank you for stating the following in your Competing Interests section:

"NO authors have competing interests"

Please complete your Competing Interests on the online submission form to state any Competing Interests. If you have no competing interests, please state "The authors have declared that no competing interests exist.", as detailed online in our guide for authors at http://journals.plos.org/plosone/s/submit-now

This information should be included in your cover letter; we will change the online submission form on your behalf.

5. When completing the data availability statement of the submission form, you indicated that you will make your data available on acceptance. We strongly recommend all authors decide on a data sharing plan before acceptance, as the process can be lengthy and hold up publication timelines. Please note that, though access restrictions are acceptable now, your entire data will need to be made freely accessible if your manuscript is accepted for publication. This policy applies to all data except where public deposition would breach compliance with the protocol approved by your research ethics board. If you are unable to adhere to our open data policy, please kindly revise your statement to explain your reasoning and we will seek the editor's input on an exemption. Please be assured that, once you have provided your new statement, the assessment of your exemption will not hold up the peer review process.

6. If the reviewer comments include a recommendation to cite specific previously published works, please review and evaluate these publications to determine whether they are relevant and should be cited. There is no requirement to cite these works unless the editor has indicated otherwise.

[Note: HTML markup is below. Please do not edit.]

Reviewers' comments:

Reviewer's Responses to Questions

**Comments to the Author**

1. Is the manuscript technically sound, and do the data support the conclusions?

Reviewer #1: Yes

Reviewer #2: No

**********

2. Has the statistical analysis been performed appropriately and rigorously?

Reviewer #1: Yes

Reviewer #2: I Don't Know

**********

3. Have the authors made all data underlying the findings in their manuscript fully available?

The PLOS Data policy

Reviewer #1: Yes

Reviewer #2: No

**********

4. Is the manuscript presented in an intelligible fashion and written in standard English?

Reviewer #1: Yes

Reviewer #2: Yes

**********

Reviewer #1: The manuscript "Population differences in embryonic developmental timing in the lizard Diploderma swinhonis" provides interesting information about the embryology and ecology of the species. This manuscript has potential for publication; however, some corrections need to be made. Below are some considerations regarding the text:

- Summary – Add the methodology.

- The introduction is well-written and leads the reader to the central theme of the research. I suggest only adding the distance from the orchid island to the mainland of Taiwan.

- Methodology –

Line 102: State the average incubation period of the species' eggs.

Lines 119-120: Was the first embryo collected immediately after oviposition? And then at 10-day intervals? Make this clearer in the text. Why was this collection interval chosen?

How many collections were there in total? Add this information.

Line 121: “according to a standardized developmental table.” Include the reference for this standard table.

It needs to be explained that the images used to present embryonic development were of embryos from mainland females.

- Results

Don't you think the laboratory environment influenced the time and stages of embryonic development after all the eggs were placed in the same environmental condition?

Please explain in the text which stage the embryo was in at the time of oviposition in each location. And also which stage in the embryos collected at 10-day intervals.

Figure 1 should be changed as it is out of focus.

Figure 2 – At stage 30 I can already identify the mandibular process.

Line 262: “Stage 35” – I believe this refers to stages 35 to 38. Because the description below covers these three stages.

Line 310: “In the stage 41” – the text refers to stage 42.

- Discussion

From lines 313 to 325 the text was merely a repetition of the results. The discussion should interpret and explain the meaning of the results obtained, not just repeat them. In addition to relating them to other studies already carried out.

Lines 337 and 348 describe the same information: "This pattern is consistent with the concept of facultative egg retention."

Information about embryonic development needs to be added to the discussion.

Reviewer #2: The article aims to compare the embryonic development of eggs laid by lizard females collected on Taiwan’s main island to that of eggs laid by lizard females collected on Orchid Island. The abstract and introduction are very well written, and the topic is interesting. Unfortunately, the article suffers from a lack of precision and organization, which makes it difficult to assess the validity of the conclusions. Below are some comments that should be taken into account to improve the article. As it stands, it is difficult to determine how the experimental data were used to develop the equations.

I suggest including a workflow of the experiments conducted, as I have difficulty understanding the various steps. Below are listed numerous details need to be included.

Regarding the “experiments” section, I have several questions:

When collecting the lizards, how do you distinguish the females from the males?

How long does incubation last until hatching?

According to the Methods section, eggs were opened every 10 days and the embryos were fixed. What is the protocol? What was the size/weight of the eggs? How was the dissection performed, given that I assume that the eggs are very small? How many eggs were analysed every ten days, for both groups?

It would also be helpful to include a summary table of the number of clutches per female and the size of the clutches for each females.

Presenting the equations and prediction models before even showing the obtained images is quite unusual.

To better appreciate the conclusion stating that eggs laid by lizards on Orchid Island are at a more advanced stage of development than those laid by females on the main island—which implies longer egg retention in the female— a comparison of embryonic stages at oviposition and a figure showing the obtained photos would be more informative (while including the number of replicates).

The figures included in the main text of the article are those obtained for the lizard from the main island, but the lack of a visual comparison with those of the lizard from Orchid Island (which are relegated to the supplementary data) makes it impossible to appreciate the differences. As they stand, the figures in the article resemble an atlas of embryonic development and do not provide any original information compared to what is available in the literature. Similarly, the presentation of the results and their analysis lack clarity, as the equations provided to predict development and hatching synchrony are too complicated for non-experts.

It would have been interesting to weigh the lizard females and embryos at each stage and see whether there is a difference between the two groups.

The incubation of eggs have been performed under standardized conditions but difference of temperature between the two geographical regions may also play a role in the retention in females and kinetics of development. You do not discuss this aspect.

Minor remarks:

I suggest moving lines 96-104 to the introduction section.

In Figure 1, there are a A and B that lack description in the figure legend.

Supporting information 10: Figure S1.I-L. The stage 39 appears twice (I and J).

**********

what does this mean?). If published, this will include your full peer review and any attached files.

If you choose “no”, your identity will remain anonymous but your review may still be made public.

**Do you want your identity to be public for this peer review?**  For information about this choice, including consent withdrawal, please see our Privacy Policy

Reviewer #1: No

Reviewer #2: No

**********

[NOTE: If reviewer comments were submitted as an attachment file, they will be attached to this email and accessible via the submission site. Please log into your account, locate the manuscript record, and check for the action link "View Attachments". If this link does not appear, there are no attachment files.]

To ensure your figures meet our technical requirements, please review our figure guidelines: https://journals.plos.org/plosone/s/figures

You may also use PLOS’s free figure tool, NAAS, to help you prepare publication quality figures: https://journals.plos.org/plosone/s/figures#loc-tools-for-figure-preparation.

NAAS will assess whether your figures meet our technical requirements by comparing each figure against our figure specifications.

# Author comment: Author response to Decision Letter 1

**DOI:** 10.1371/journal.pone.0359078.r002

24 Jun 2026

Response to Reviewers

Manuscript ID: ONE-D-26-13257

Title: Population differences in embryonic developmental timing in the lizard Diploderma swinhonis

Dear Academic Editor and Reviewers,

We sincerely thank the Academic Editor and both reviewers for their constructive comments and valuable suggestions. We have carefully revised the manuscript according to all comments received.

All changes in the revised manuscript are highlighted in the tracked-changes version.

Detailed responses to each comment are provided below.

Responses to the Academic Editor

Journal Requirements:

Requirement 1:

Please ensure that your manuscript meets PLOS ONE's style requirements, including those for file naming. The PLOS ONE style templates can be found at

https://journals.plos.org/plosone/s/file?id=wjVg/PLOSOne_formatting_sample_main_body.pdf and

https://journals.plos.org/plosone/s/file?id=ba62/PLOSOne_formatting_sample_title_authors_affiliations.pdf

Response: Thanks for your reminder. We have updated the formatting of our manuscript to meet PLOS ONE’s style requirements.

(Line 104-117)

Requirement 2:

To comply with PLOS One submissions requirements, in your Methods section, please provide additional information regarding the experiments involving animals and ensure you have included details on (1) methods of sacrifice, (2) methods of anesthesia and/or analgesia, and (3) efforts to alleviate suffering.

Response: Thanks for your suggestion. We have updated the Methods section to provide detailed information regarding the animal experiments, including the methods of sacrifice, and the efforts made to alleviate suffering.

“All experimental procedures were conducted in accordance with institutional animal care guidelines and approved animal use protocols. According to the Guide for the Care and Use of Laboratory Animals issued by the Ministry of Agriculture [34], the guiding principles for handling reptile embryos and hatchlings to minimize pain are as follows: Early Incubation Stage (Development < 20%): The nervous system is not yet fully developed, so they are generally considered insensible to pain. Unhatched eggs or those in early developmental stages can be directly frozen to achieve euthanasia. Late Incubation Stage (Development > 20%): The embryonic nervous system is more mature, making them capable of experiencing pain and distress. Regulations require breaking the eggshell to remove the embryo. Pain must be mitigated through chemical methods, such as anesthetic injections (e.g., Pentobarbital), or physical methods, such as decapitation. Lizard hatchlings must receive the same treatment as mature individuals. They must undergo standard chemical anesthesia or physical euthanasia; under no circumstances shall direct freezing be permitted.”

Requirement 3:

Thank you for stating the following financial disclosure:

"MOST 113-2621-B-178-001- MY3"

Please state what role the funders took in the study. If the funders had no role, please state: "The funders had no role in study design, data collection and analysis, decision to publish, or preparation of the manuscript."

If this statement is not correct you must amend it as needed.

Please include this amended Role of Funder statement in your cover letter; we will change the online submission form on your behalf.

Response: Thanks for your reminder. We confirm that the statement provided by the journal is entirely correct. The funders had no role in study design, data collection and analysis, decision to publish, or preparation of the manuscript.

As requested, we have included this exact "Role of Funder" statement in our revised Cover Letter.

Requirement 4: Thank you for stating the following in your Competing Interests section: "NO authors have competing interests" Please complete your Competing Interests on the online submission form to state any Competing Interests. If you have no competing interests, please state "The authors have declared that no competing interests exist.", as detailed online in our guide for authors at http://journals.plos.org/plosone/s/submit-now

This information should be included in your cover letter; we will change the online submission form on your behalf.

Response: Thank you for the reminder. We have no competing interests and have included the exact statement: "The authors have declared that no competing interests exist."

Requirement 5:

When completing the data availability statement of the submission form, you indicated that you will make your data available on acceptance. We strongly recommend all authors decide on a data sharing plan before acceptance, as the process can be lengthy and hold up publication timelines. Please note that, though access restrictions are acceptable now, your entire data will need to be made freely accessible if your manuscript is accepted for publication. This policy applies to all data except where public deposition would breach compliance with the protocol approved by your research ethics board. If you are unable to adhere to our open data policy, please kindly revise your statement to explain your reasoning and we will seek the editor's input on an exemption. Please be assured that, once you have provided your new statement, the assessment of your exemption will not hold up the peer review process.

Response: Thanks for your suggestions. We have revised the Data Availability Statement and clarified our data sharing plan. Data are available to editors and reviewers during peer review through a Figshare review link and will remain publicly accessible following publication.

“All data underlying the findings of this study are available in Figshare at https://doi.org/10.6084/m9.figshare.30304954.v1.”

Requirement 6:

If the reviewer comments include a recommendation to cite specific previously published works, please review and evaluate these publications to determine whether they are relevant and should be cited.

Response: Relevant references have been incorporated into the revised manuscript where appropriate.

Responses to Reviewer #1

We thank Reviewer #1 for the positive evaluation and constructive suggestions.

(Line 20-25)

Comment 1:

Summary – Add the methodology.

Response: As suggested, we have expanded the Abstract to include the methodology used in this study.

“Gravid females from mainland Taiwan and Orchid Island were collected and maintained under standardized laboratory conditions. Following oviposition, eggs within each clutch were sequentially dissected at 10-day intervals to minimize maternal effects and avoid pseudoreplication. Embryonic developmental stages were determined using a standardized developmental staging table, and developmental trajectories were compared between populations.”

(Lines 77-79)

Comment 2:

Introduction

Add the distance from Orchid Island to mainland Taiwan.

Response: As suggested, we have specified the distance in the Introduction, noting that Orchid Island is located approximately 63 to 72 kilometers off the southeast coast of mainland Taiwan.

(Line 146-149)

Comment 3:

Methodology

Line 102: State the average incubation period of the species' eggs.

Response: As suggested, we have specified the egg incubation period in the Methodology section. We noted that under our laboratory conditions, the average incubation period of D. swinhonis eggs was approximately 48-49 days.

(Line 144-156)

Comment 4:

Methodology

Lines 119-120: Was the first embryo collected immediately after oviposition? And then at 10-day intervals? Make this clearer in the text. Why was this collection interval chosen? How many collections were there in total? Add this information. Line 121: “according to a standardized developmental table.” Include the reference for this standard table.

Response: Thank you for these excellent and detailed questions. We have thoroughly revised the Methodology section to clarify the egg collection protocol, the rationale behind the 10-day sampling interval, the total number of collections, and the reference for the developmental staging table. These comprehensive details have been added to the Methodology section. For your convenience, the updated text is provided below:

“Gravid females were collected approximately 5-6 times, and females typically produced clutches of three to four eggs. Eggs were collected immediately after oviposition and assigned to predetermined dissection dates. The 10-day interval was selected because embryonic development in this species spans approximately 48-49 days, allowing major developmental transitions to be captured across incubation while ensuring that developmental sampling was distributed throughout the entire incubation period. For example, if the first egg from a clutch was dissected on a given sampling day, the remaining eggs from that clutch were dissected 10, 20, 30, and 40 days later, respectively. This sequential within-clutch dissection design was used to avoid repeatedly dissecting multiple eggs from the same female on the same day, thereby minimizing maternal effects and reducing pseudoreplication in developmental time-series analyses. Embryos were subsequently dissected and staged according to the developmental staging table of Dufaure and Hubert (1961) [6], originally developed for lacertid lizards and adapted here for staging embryonic development in D. swinhonis.”

(Figure 2-5)

Comment 5:

It needs to be explained that the images used to present embryonic development were of embryos from mainland females.

Response: Thank you for pointing this out. To maximize clarity and facilitate direct visual comparison between the two populations, we have updated Figure 2-5. Representative embryos from mainland Taiwan (upper row) and Orchid Island (lower row) are now shown side-by-side. The figure legend has been revised accordingly.

Comment 6:

- Results

Don't you think the laboratory environment influenced the time and stages of embryonic development after all the eggs were placed in the same environmental condition?

Response: We completely agree with the reviewer that the incubation environment plays a critical role in embryonic development. Indeed, environmental temperature and moisture typically exert strong influences on developmental rates in reptiles. However, the fact that population-level differences persisted despite all eggs being maintained under identical, standardized laboratory conditions is one of the key findings of our study.

If the post-ovipositional environment were the sole determinant of developmental timing, both populations should have displayed identical trajectories and highly synchronized development under the same laboratory conditions. Instead, our data revealed distinct, population-specific traits.

(Line 199-205)

Comment 7:

Please explain in the text which stage the embryo was in at the time of oviposition in each location. And also which stage in the embryos collected at 10-day intervals.

Response: Thank you for this suggestion. We have revised the Results section to explicitly state the embryonic developmental stages at the time of oviposition for both locations, as well as the progressive stages observed at each 10-day sampling interval. For your convenience, the updated text reads as follows:

“We observed that embryos from mainland Taiwan were generally deposited at stages 27-28, whereas embryos from Orchid Island were deposited at the more advanced stage 31. Across incubation, embryos sampled at approximately 10-day intervals showed progressive developmental advancement, with embryos reaching approximately stages 33-34 after 10 days, 36 after 20 days, 39 after 30 days, and 41-42 after 40 days. Representative embryonic images from both populations were included to facilitate direct visual comparison of developmental stages between populations.”

(Figure 6)

Comment 8:

Figure 1 is out of focus.

Response: As suggested, Figure 6 (Figure 1 is changed to Figure 6) has been replaced with a higher-resolution image reaching 600 ppi.

(Figure 3)

Comment 9:

Figure 2 – At stage 30 I can already identify the mandibular process.

Response: Thank you for the observation. We believe there might be a slight misunderstanding regarding the structures. In Stage 30 (Figure 2 is changed to Figure 3), the visible lateral structures are actually the pharyngeal clefts ("The first through fifth pharyngeal clefts were clearly visible"), as described in manuscript (Line 231). According to the criteria of Dufaure and Hubert (1961), the mandibular process (mdp) in our specimens becomes distinct and is formally labeled later at Stage 32 (as shown in Figure 3). Therefore, based on the overall suite of diagnostic characters, the specimen remains accurately staged as Stage 30.

Comment 10:

- Discussion

1. Line 262: “Stage 35” – I believe this refers to stages 35 to 38. Because the description below covers these three stages.

2. Line 310: “In the stage 41” – the text refers to stage 42.

Line 260

Response to Line 262: Thank you for pointing this out. We have corrected "Stage 35" to "stages 35-38".

Line 288

Response to Line 310: Thank you for your careful reading. We have corrected the typographical error and changed "In the stage 41" to "In stage 39-42" to correctly match the referenced text.

(Line 358-431)

Comment 11:

- Discussion

From lines 313 to 325 the text was merely a repetition of the results. The discussion should interpret and explain the meaning of the results obtained, not just repeat them. In addition to relating them to other studies already carried out. Lines 337 and 348 describe the same information: "This pattern is consistent with the concept of facultative egg retention." Information about embryonic development needs to be added to the discussion.

Response: As suggested, these revisions substantially changed the Discussion from a descriptive summary of results to a more interpretation-driven framework.

“The present study demonstrates clear population-level differences in embryonic developmental timing in D. swinhonis. Eggs from Orchid Island were deposited at significantly more advanced developmental stages than those from mainland Taiwan, indicating prolonged intra-oviductal egg retention in the Orchid Island population. Because embryos from both populations were subsequently maintained under identical laboratory conditions, the observed differences are unlikely to result solely from post-oviposition environmental effects. Instead, these findings suggest divergence in reproductive timing and maternal regulation of embryonic development between geographically isolated populations.

Developmental staging further revealed that embryos from both populations followed broadly similar morphological trajectories during incubation, indicating that the primary difference lies in the timing of oviposition rather than major divergence in developmental patterning itself. Thus, the populations appear to differ mainly in developmental timing (heterochrony) rather than developmental sequence [40]. Such heterochronic shifts may represent an adaptive mechanism that allows populations to respond to local ecological pressures while maintaining conserved developmental pathways.

Developmental synchrony significantly differed between populations, with Orchid Island embryos exhibiting greater individual variation post-oviposition. It suggests that prolonged maternal egg retention may fundamentally alter intrinsic developmental dynamics. From an evolutionary perspective, this lower synchrony may serve as an adaptive bet-hedging strategy under intense insular predation pressure. Reptilian embryos at late stages exhibit peak metabolic rates, releasing significant volatile organic compounds that create a concentrated "olfactory target" for specialized egg-eating predators, such as O formosanus on Orchid Island [31, 41]. Conversely, asynchronous development temporally staggers these individual metabolic peaks, potentially diluting the overall chemical signal to evade detection. Furthermore, such asynchrony naturally extends the hatching window among siblings, reducing the risk of cohort-wide mortality or synchronized predator swarming during emergence. Therefore, prolonged retention may function as a multi-layered reproductive strategy shaping subsequent embryogenesis to maximize offspring survival in high-risk ecosystems.

Standardized maternal body size showed no detectable effect on developmental rate, suggesting that variation in embryonic timing is unlikely to be explained simply by female morphology. Instead, the observed population differences are more consistent with ecological influences on reproductive timing. Such patterns are consistent with facultative egg retention [42, 43], in which females adjust the duration of intra-oviductal embryo retention in response to local ecological conditions. Facultative egg retention has been documented in several squamate reptiles and is frequently interpreted as an intermediate reproductive strategy along the evolutionary continuum between oviparity and viviparity [44, 45]. The contrasting developmental patterns observed between Orchid Island and mainland Taiwan populations therefore suggest that geographic variation in ecological conditions may shape maternal control over embryonic development and reproductive timing.

The distinctive embryonic developmental pattern observed in the Orchid Island population may also be associated with the unique ecological context of this island, particularly the exceptionally high predation pressure imposed by the egg-eating snake O. formosanus. Previous studies have demonstrated that Orchid Island supports unusually high densities of this predator due to the seasonal availability of sea turtle nests, which subsequently increases predation pressure on reptile eggs, including those of lizards. Strong egg predation pressure on Orchid Island has already been linked to several remarkable evolutionary responses in insular lizards. For example, Orchid Island populations of the long-tailed skink (Eutropis longicaudata) exhibit post-ovipositional maternal care in the form of nest guarding behavior, a trait absent in mainland populations [30]. Experimental studies further demonstrated that this maternal care behavior evolved specifically as a defense against egg predation by O. formosanus. In addition, Orchid Island skinks exhibit significantly greater bite force than mainland populations, likely as an adaptive response associated with parental defense and predator deterrence.

Environmental temperature is known to influence developmental rates and reproductive traits in reptiles. However, mainland Taiwan and Orchid Island occur at similar latitudes and share broadly comparable climatic conditions and habitats, reducing the likelihood that large-scale environmental temperature differences alone explain the observed developmental divergence. Nevertheless, we cannot exclude the possibility that fine-scale environmental variation contributes to population differences in reproductive timing and egg retention.

When integrated into this ecological context, the advanced embryonic stages observed at oviposition in Orchid Island D. swinhonis may represent another reproductive response to intense egg predation pressure. Prolonged intra-oviductal egg retention could reduce the duration of vulnerable external incubation, thereby shortening the period during which eggs remain exposed to predators. Although the present study does not directly test the adaptive fitness consequences of egg retention, the observed developmental divergence is consistent with the hypothesis that predator-driven selection on Orchid Island has shaped multiple reproductive and developmental traits in reptiles inhabiting this insular ecosystem. More broadly, these findings support the idea that reproductive timing, parental investment, and embryo retention can evolve rapidly under strong ecological pressures. The Orchid Island population therefore provides a valuable natural system for understanding how localized predation pressure may influence the evolution of reproductive strategies and embryonic developmental timing in oviparous reptiles.”

Responses to Reviewer #2

We thank Reviewer #2 for the detailed and thoughtful comments, which significantly improved the organization and clarity of the manuscript.

Comment 1:

As it stands, it is difficult to determine how the experimental data were used to develop the equations.

Response: Thank you for your comment. We have simplified the statistical analyses section while maintaining its accuracy.

(Figure 1)

Comment 2:

I suggest including a workflow of the experiments conducted, as I have difficulty understanding the various steps. Below are listed numerous details need to be included.

Response: We completely agree that a visual workflow is highly beneficial for clarifying the experimental timeline. As suggested, we have created a comprehensive schematic diagram from and integrated it directly into the main text as Figure 1 at the beginning of the Materials and Methods section.

This figure explicitly outlines all experimental phases requested, including field sampling, laboratory housing, the sequential within-clutch 10-day egg dissection design, embryo staging, and statistical analyses.

Comment 3:

When collecting the lizards, how do you distinguish the females from the males?

Response: Thank you for this question. Lizards were collected during the breeding season when gravid females are reproductively active. Sexes were distinguished based on external morphology. Males are generally larger and possess broader heads, longer limbs, brighter coloration, and distinct yellow flank stripes. Females are typically smaller, duller in coloration, and exhibit more slender body shapes. Behavioral differences were also used as supplementary cues, with males more frequently perching higher on tree trunks and females occurring closer to the ground within vegetation.

(Line 146-149)

Comment 4:

How long does incubation last until hatching?

Response: Thank you for this question. Under our laboratory conditions, the average incubation period of D. swinhonis eggs was approximately 48-49 days.

(Line 144-156)

Comment 5:

According to the Methods section, eggs were opened every 10 days and the embryos were fixed. What is the protocol?

Response: Thank you for this question. We have updated the Methods section and the specific protocol text reads as follows:

“Gravid females were captured in the field and maintained under controlled laboratory conditions until oviposition. During the breeding season, gravid females were collected approximately 5-6 times, and females typically produced clutches of three to four eggs. Eggs were collected immediately after oviposition and assigned to predetermined dissection dates. The 10-day interval was selected because embryonic development in this species spans approximately 48-49 days, allowing major developmental transitions to be captured across incubation while ensuring that developmental sampling was distributed throughout the entire incubation period. For example, if the first egg from a clutch was dissected on a given sampling day, the remaining eggs from that clutch were dissected 10, 20, 30, and 40 days later, respectively. This sequential within-clutch dissection design was used to avoid repeatedly dissecting multiple eggs from the same female on the same day, thereby minimizing maternal effects and reducing pseudoreplication in developmental time-series analyses. Embryos were subsequently dissected and staged according to the developmental staging table of Dufaure and Hubert (1961), originally developed for lacertid lizards and adapted here for staging embryonic development in D. swinhonis.

Comment 6:

What was the size/weight of the eggs?

Response: Thank you for this critical point. We agree that it is important to clarify the source of these morphological data. These measurements were obtained from previous records of the same populations prior to the current experiment, rather than from the exact clutches used for embryonic staging in this study.

Following oviposition, egg length (mm), egg width (mm), and egg mass (g) from previous breeding records are presented here to characterize egg morphology. Eggs from mainland Taiwan measured 12.77 ± 0.81 mm in length, 7.60 ± 0.73 mm in width, and weighed 0.442 ± 0.073 g, whereas eggs from Orchid Island measured 13.71 ± 1.17 mm in length, 8.69 ± 1.64 mm in width, and weighed 0.593 ± 0.181 g (mean ± SD).

(Line 159-163)

Comment 7:

How was the dissection performed, given that I assume that the eggs are very small?

Response: Thank you for this comment. We have clarified the dissection procedure in the revised manuscript.

“Egg dissections were performed under a stereomicroscope using fine dissection scissors and forceps. The eggshell was carefully opened, and surrounding extraembryonic tissues were gradually removed to expose the embryo for developmental staging. All dissections were conducted carefully to minimize mechanical damage to embryonic structures.”

Comment 8:

How many eggs were analysed every ten days, for both groups?

Response: Thank you for this comment. To provide full transparency regarding our sample sizes across the time-series, we have compiled these details into a new table and integrated it directly into the main text as Table 1 within the Materials and Methods section (subsection: Embryo Collection and Embryo developmental stages Analyses). As shown in Table 1, a total of 132 embryos from Orchid Island and 132 embryos from mainland Taiwan were analyzed across the sequential developmental intervals, demonstrating an identical and balanced sampling effort between the two groups:

Number of D. swinhonis embryos analyzed across developmental intervals.

Developmental interval (days) Orchid Island (n) Mainland Taiwan (n)

Days 0-9 40 40

Days 10-19 34 34

Days 20-29 20 20

Days 30-39 20 20

Days 40-48 18 18

Total embryos analyzed 132 132

Comment 9:

It would also be helpful to include a summary table of the number of clutches per female and the size of the clutches for each female.

Response: Thank you for this suggestion. Each gravid female captured for this study contributed only a single clutch to the analyses. Following oviposition, all females were promptly returned and released at their original field collection sites to minimize ecological disturbance and reduce the duration of captivity.

Comment 10:

Presenting the equations and prediction models before even showing the obtained images is quite unusual.

Response: We sincerely apologize for the unconventional presentation in the previous version. We fully agree with the reviewer that presenting abstract equations and model predictions prior to describing the actual empirical baseline data (the obtained embryonic images and morphological staging) is logically counterintuitive. To correct this, we have thoroughly reorganized the Materials and Methods and Results sections.

Comment 11:

1. To better appreciate the conclusion stating that eggs laid by lizards on Orchid Island are at a more advanced stage of development than those laid by females on the main island—which implies longer egg retention in the female— a comparison of embryonic stages at oviposition and a figure showing the obtained photos would be more informative (while including the number of replicates).

2. The figures included in the main text of the article are those obtained for the lizard from the main island, but the lack of a visual comparison with those of the lizard from Orchid Island (which are relegated to the supplementary data) makes it impossible to appreciate the differences. As they stand, the figures in the article resemble an atlas of embryonic development and do not provide any original information compared to what is available in the literature.

Response:

We completely agree with the reviewer that providing a direct, comprehensive visual comparison across the entire developmental spectrum—rather than limiting the Orchid Island series to the supplementary data—is fundamental to showcasing the population-level divergence. We realize that our previous figure arrangement resembled a descriptive "atlas" for a single population, which obscured the dynamic heterochrony and altered synchrony between mainland Taiwan and Orchid Island embryos.

To address this comprehensively across the entire developmental process, we have implemented two major structural enhancements in the revised manuscript:

1. Integration of Figure 6 (Full Time-Series Empirical Data and Trajectories):

� This figure provides an immediate, population-level visual comparison of the entire post-oviposition timeline (N = 264 total embryos).

� It explicitly highlights the initial gap at oviposition (Day 0), where mainland embryos exhibit high variability across early Stages 27–29 (n = 4 for each stage), whereas Orchid Island embryos initiate development at an advanced, tightly clustered baseline of Stages 31–32 (n = 4 for each stage).

Crucially, the figure tracks how these developmental trajectories dynamically diverge and shift across the entire incubation period, validated by our Bayesian cumulative ordinal regression model.

2. Complete Restructuring of Embryonic Series Layout (Figures 2 to 5):

To facilitate direct morphological comparison throughout embryogenesis as

requested, we have completely overhauled the layout of our main-text

figures:

� Figure 2 (Stages 27–30): This figure features only a single row representing the mainland Taiwan population. This accurately reflects our empirical findings (as shown in Figure 6); no Orchid Island embryos were ever captured at these early stages because their oviposition window consistently initiates at Stage 31.

� Figures 3, 4, and 5 (Stages 31–42): These figures have been reorganized from separate population views into a side-by-side comparative matrix consisting of two parallel rows (Row A: mainland Taiwan; Row B: Orchid Island). This alignment allows readers and reviewers to easily track and contrast the synchronized morphological progression, visually demonstrating how prolonged egg retention fundamentally reshapes subsequent developmental dynamics throughout the entire process.

Comment 12:

Similarly, the presentation of the results and their analysis lack clarity, as the equations provided to predict development and hatching synchrony are too complicated for non-experts

Response: We appreciate your suggestion. We have revised the Results section by reducing complex statistical terminology and removing repeated sentences to improve clarity and readability for a broader audience.

Comment 13:

It would have been interesting to weigh the lizard females and embryos at each stage and see whether there is a difference between the two groups.

Response: Thank you for this valuable suggestion. Female body mass was not systematically recorded in the present study because the primary objective was to examine population differences in embryonic developmental timing rather than variation in maternal morphology. Instead, maternal body size was standardized using morphological measurements to account for potential maternal effects. We agree that incorporating female body mass and embryo mass across developmental stages could provide additional insight into maternal investment and developmental trajectories and consider this an important direction for future studies.

(Line 413-418)

Comment 14:

The incubation of eggs have been performed under standardized conditions but difference of temperature between the two geographical regions may also play a role in the retention in females and kinetics of development. You do not discuss this aspect.

Response: Thank you for this important comment. We agree that environmental temperature may influence embryonic development and egg retention in reptiles. However, mainland Taiwan and Orchid Island occur at similar latitudes and share broadly comparable climatic conditions and habitat characteristics, which was one reason these populations were selected for comparison. Furthermore, all eggs were incubated under standardized laboratory conditions after oviposition, minimizing post-ovipositional environmental effects on embryonic development.

Therefore, although we cannot completely exclude the possibility that local thermal environments contribute to population divergence, the observed differences in developmental stage at oviposition are more likely to reflect differences occurring prior to oviposition, including variation in maternal reproductive timing and egg retention. We have added this discussion to the revised manuscript.

“Environmental temperature is known to influence developmental rates and reproductive traits in reptiles. However, mainland Taiwan and Orchid Island occur at similar latitudes and share broadly comparable climatic conditions and habitats, reducing the likelihood that large-scale environmental temperature differences alone explain the observed developmental divergence. Moreover, because all eggs were incubated under standardized laboratory conditions following oviposition, differences observed during incubation are more likely to reflect developmental processes occurring prior to oviposition. Nevertheless, we cannot exclude the possibility that fine-scale environmental variation contributes to population differences in reproductive timing and egg retention.”

(Line 73-79)

Comment 15:

Minor remarks:

I suggest moving lines 96-104 to the introduction section.

Response: Thank you for this excellent suggestion. As recommended, we have moved the indicated text (previously lines 96-104) from the Methods section to the Introduction section.

(Figure 6, Line 340-357)

Comment 16:

In Figure 1, there are a A and B that lack description in the figure legend.

Response: We apologize for this oversight. We have thoroughly revised the figure legend to provide explicit, detailed descriptions for both Panel A and Panel B to ensure complete clarity for the readers.

The figure legend has been updated in the revised manuscript as follows: Figure 6. Posterior predictions of embryonic developmental trajectories and empirical data distributions for mainland Taiwan (TW) and Orchid Island (OI) populations of Diploderma swinhonis.

“(A) Empirical sample distributions and stage proportions across sampling days: This panel presents the raw empirical distribution of sampled embryos. The upper grid represents mainland Taiwan (TW) and the lower grid represents Orchid Island (OI). Circle size (N) indicates the raw number of embryos dissected on that specific post-oviposition day (ranging from 2 to 4). The color coding of the circles represents the relative proportion of individuals at that specific developmental stage within that day's sample. The numerical values above the symbols indicate the total replicate size (N) for clearer reference.

(B) Bayesian model posterior predictions and trajectories: This panel displays the estimated continuous developmental trajectories derived from our Bayesian cumulative ordinal regression model. Blue hollow circles and lines represent the mainland Taiwan population (TW), while yellow filled circles and lines represent the Orchid Island population (OI). The solid trend lines represent the most probable embryonic stage predicted for each post-oviposition day. The size of the circles indicates the posterior probability (ranging from 0.1 to 0.9) of an embryo being at a given stage on a specific day, clearly demonstrating that Orchid Island embryos initiate development earlier, progress more slowly, and exhibit lower developmental synchrony.”

Comment 18:

Supporting information 10: Figure S1.I-L. The stage 39 appears twice (I and J).

Response: Thank you for catching this error. We have corrected the labeling mistake and completely restructured the figure layout.

These developmental stages have been moved from the Supporting Information into the main text as Figure 5. In this revised figure, representative embryonic photos from mainland Taiwan and Orchid Island are placed together side-by-side to facilitate a direct visual comparison across synchronized stages.

Attachment Submitted filename: 2. Response to reviewers (1).docx

# Peer review: Decision Letter 1

**DOI:** 10.1371/journal.pone.0359078.r003

**Authors:** Renato Filogonio

19 Aug 2026

Dear Dr. Huang,

Thank you for submitting your manuscript to PLOS One. After careful consideration, we feel that it has merit but does not fully meet PLOS One’s publication criteria as it currently stands. Therefore, we invite you to submit a revised version of the manuscript that addresses the points raised during the review process.

==============================

**ACADEMIC EDITOR:**

While I understand that this version was greatly improved, Reviewer 3 suggests that authors should consider the differences in climatic conditions between sites, if they exist. This is briefly mentioned in the introduction and I agree with the reviewer´s assessment that this should not be overlooked.

Additionally, I have two minor points to add:

1) Lines 112-116: Please indicate the exact method of anesthesia utilized during euthanasia, indicating which anesthetic was used, procedence (supplier, country) and the dose used.

2) Lines 150-151: The authors mention that the eggs were dissected at sampling day (day 0), than again at days 10, 20, 30 and 40. This accounts for a total of 5 eggs, although the authors inform that clutches had 3-4 eggs (line 145). Additionally, in the new Figure 1, the authors indicate that eggs were dissected only until day 30. Please correct accordingly.

==============================

Please submit your revised manuscript by Oct 03 2026 11:59PM. If you will need more time than this to complete your revisions, please reply to this message or contact the journal office at plosone@plos.org. When you're ready to submit your revision, log on to https://www.editorialmanager.com/pone/ and select the 'Submissions Needing Revision' folder to locate your manuscript file.

Please include the following items when submitting your revised manuscript:

- A letter that responds to each point raised by the academic editor and reviewer(s). You should upload this letter as a separate file labeled 'Response to Reviewers'.
- A marked-up copy of your manuscript that highlights changes made to the original version. You should upload this as a separate file labeled 'Revised Manuscript with Track Changes'.
- An unmarked version of your revised paper without tracked changes. You should upload this as a separate file labeled 'Manuscript'.

If you would like to make changes to your financial disclosure, please include your updated statement in your cover letter. Guidelines for resubmitting your figure files are available below the reviewer comments at the end of this letter.

If applicable, we recommend that you deposit your laboratory protocols in protocols.io to enhance the reproducibility of your results. Protocols.io assigns your protocol its own identifier (DOI) so that it can be cited independently in the future. For instructions see: https://journals.plos.org/plosone/s/submission-guidelines#loc-laboratory-protocols. Additionally, PLOS One offers an option for publishing peer-reviewed Lab Protocol articles, which describe protocols hosted on protocols.io. Read more information on sharing protocols at https://plos.org/protocols?utm_medium=editorial-email&utm_source=authorletters&utm_campaign=protocols.

As the corresponding author, your ORCID iD is verified in the submission system and will appear in the published article. PLOS supports the use of ORCID, and we encourage all coauthors to register for an ORCID iD and use it as well. Please encourage your coauthors to verify their ORCID iD within the submission system before final acceptance, as unverified ORCID iDs will not appear in the published article. *Only*  the individual author can complete the verification step; PLOS staff *cannot*  verify ORCID iDs on behalf of authors.

We look forward to receiving your revised manuscript.

Kind regards,

Renato Filogonio, Ph.D.

Academic Editor

PLOS One

Journal Requirements:

If the reviewer comments include a recommendation to cite specific previously published works, please review and evaluate these publications to determine whether they are relevant and should be cited. There is no requirement to cite these works unless the editor has indicated otherwise.

[Note: HTML markup is below. Please do not edit.]

Reviewers' comments:

Reviewer's Responses to Questions

**Comments to the Author**

Reviewer #1: (No Response)

Reviewer #3: (No Response)

**********

2. Is the manuscript technically sound, and do the data support the conclusions?

Reviewer #1: Yes

Reviewer #3: Partly

**********

3. Has the statistical analysis been performed appropriately and rigorously?

Reviewer #1: Yes

Reviewer #3: Yes

**********

4. Have the authors made all data underlying the findings in their manuscript fully available?

The PLOS Data policy

Reviewer #1: Yes

Reviewer #3: Yes

**********

5. Is the manuscript presented in an intelligible fashion and written in standard English?

Reviewer #1: Yes

Reviewer #3: Yes

**********

Reviewer #1: I appreciate the authors' responses regarding the manuscript "Population differences in embryonic developmental timing in the lizard *Diploderma swinhonis*." The suggestions and considerations have been incorporated into the text, which has improved the work and made it suitable for publication.

Reviewer #3: The study is interesting, and the information it provides is presented in a clear and concise manner. However, I believe that the study should not overlook the climatic conditions under which these females live (both on Orchid Island and in Taiwan). I think it is necessary to add to the manuscript and briefly discuss (using historical records) the climatic conditions that could be affecting both egg retention and environmental incubation. Perhaps rainfall or temperature can better explain the slower development of the embryos on Ochid Island during (external) incubation.

**********

what does this mean?). If published, this will include your full peer review and any attached files.

If you choose “no”, your identity will remain anonymous but your review may still be made public.

**Do you want your identity to be public for this peer review?**  For information about this choice, including consent withdrawal, please see our Privacy Policy

Reviewer #1: No

Reviewer #3: No

**********

[NOTE: If reviewer comments were submitted as an attachment file, they will be attached to this email and accessible via the submission site. Please log into your account, locate the manuscript record, and check for the action link "View Attachments". If this link does not appear, there are no attachment files.]

To ensure your figures meet our technical requirements, please review our figure guidelines: https://journals.plos.org/plosone/s/figures

You may also use PLOS’s free figure tool, NAAS, to help you prepare publication quality figures: https://journals.plos.org/plosone/s/figures#loc-tools-for-figure-preparation.

NAAS will assess whether your figures meet our technical requirements by comparing each figure against our figure specifications.

# Author comment: Author response to Decision Letter 2

**DOI:** 10.1371/journal.pone.0359078.r004

27 Aug 2026

no

Attachment Submitted filename: 1. Response to reviewers.docx

# Peer review: Decision Letter 2

**DOI:** 10.1371/journal.pone.0359078.r005

**Authors:** Renato Filogonio

1 Sep 2026

Dear Dr. Huang,

Thank you for submitting your manuscript to PLOS One. After careful consideration, we feel that it has merit but does not fully meet PLOS One’s publication criteria as it currently stands. Therefore, we invite you to submit a revised version of the manuscript that addresses the points raised during the review process.

==============================

**ACADEMIC EDITOR:**

The reviewer and myself were satisfied with the responses and changes to the text and commend the authors for the study. Reviewer 3 suggests one small addition to the discussion regarding the effects of the rain on the females (see below). I ask that the authors add this aspect to the discussion before accepting the manuscript.

==============================

Please submit your revised manuscript by Oct 16 2026 11:59PM. If you will need more time than this to complete your revisions, please reply to this message or contact the journal office at plosone@plos.org. When you're ready to submit your revision, log on to https://www.editorialmanager.com/pone/ and select the 'Submissions Needing Revision' folder to locate your manuscript file.

Please include the following items when submitting your revised manuscript:

- A letter that responds to each point raised by the academic editor and reviewer(s). You should upload this letter as a separate file labeled 'Response to Reviewers'.
- A marked-up copy of your manuscript that highlights changes made to the original version. You should upload this as a separate file labeled 'Revised Manuscript with Track Changes'.
- An unmarked version of your revised paper without tracked changes. You should upload this as a separate file labeled 'Manuscript'.

If you would like to make changes to your financial disclosure, please include your updated statement in your cover letter. Guidelines for resubmitting your figure files are available below the reviewer comments at the end of this letter.

If applicable, we recommend that you deposit your laboratory protocols in protocols.io to enhance the reproducibility of your results. Protocols.io assigns your protocol its own identifier (DOI) so that it can be cited independently in the future. For instructions see: https://journals.plos.org/plosone/s/submission-guidelines#loc-laboratory-protocols. Additionally, PLOS One offers an option for publishing peer-reviewed Lab Protocol articles, which describe protocols hosted on protocols.io. Read more information on sharing protocols at https://plos.org/protocols?utm_medium=editorial-email&utm_source=authorletters&utm_campaign=protocols.

As the corresponding author, your ORCID iD is verified in the submission system and will appear in the published article. PLOS supports the use of ORCID, and we encourage all coauthors to register for an ORCID iD and use it as well. Please encourage your coauthors to verify their ORCID iD within the submission system before final acceptance, as unverified ORCID iDs will not appear in the published article. *Only*  the individual author can complete the verification step; PLOS staff *cannot*  verify ORCID iDs on behalf of authors.

We look forward to receiving your revised manuscript.

Kind regards,

Renato Filogonio, Ph.D.

Academic Editor

PLOS One

Journal Requirements:

1. If the reviewer comments include a recommendation to cite specific previously published works, please review and evaluate these publications to determine whether they are relevant and should be cited. There is no requirement to cite these works unless the editor has indicated otherwise.

2. Please review your reference list to ensure that it is complete and correct. If you have cited papers that have been retracted, please include the rationale for doing so in the manuscript text, or remove these references and replace them with relevant current references. Any changes to the reference list should be mentioned in the rebuttal letter that accompanies your revised manuscript. If you need to cite a retracted article, indicate the article’s retracted status in the References list and also include a citation and full reference for the retraction notice.

[Note: HTML markup is below. Please do not edit.]

Reviewers' comments:

Reviewer's Responses to Questions

**Comments to the Author**

Reviewer #3: All comments have been addressed

**********

2. Is the manuscript technically sound, and do the data support the conclusions?

Reviewer #3: Yes

**********

3. Has the statistical analysis been performed appropriately and rigorously?

Reviewer #3: Yes

**********

4. Have the authors made all data underlying the findings in their manuscript fully available?

The PLOS Data policy

Reviewer #3: Yes

**********

5. Is the manuscript presented in an intelligible fashion and written in standard English?

Reviewer #3: Yes

**********

Reviewer #3: The study is interesting, and the results are significant. However, I find it hard to believe that the fact that it rains almost twice as much in Orchid as in Taiwan isn't just another variable affecting egg retention. And it’s very clear that it doesn’t directly affect the eggs, since they’re incubated under laboratory conditions, but it is affecting the females. I think the authors should make it sufficiently clear that it is the females who would be affected by the rains and the abundance of predators, which would cause them to retain their eggs. These Orchid eggs accelerate their embryonic development so that they hatch in the same number of days as they do in Taiwan.

I recommend publishing the study, and I would even suggest that the authors review the final table they present to see if it might be better to present it as a line or scatter plot (with the respective standard deviations).

**********

what does this mean?). If published, this will include your full peer review and any attached files.

If you choose “no”, your identity will remain anonymous but your review may still be made public.

**Do you want your identity to be public for this peer review?**  For information about this choice, including consent withdrawal, please see our Privacy Policy

Reviewer #3: No

**********

[NOTE: If reviewer comments were submitted as an attachment file, they will be attached to this email and accessible via the submission site. Please log into your account, locate the manuscript record, and check for the action link "View Attachments". If this link does not appear, there are no attachment files.]

To ensure your figures meet our technical requirements, please review our figure guidelines: https://journals.plos.org/plosone/s/figures

You may also use PLOS’s free figure tool, NAAS, to help you prepare publication quality figures: https://journals.plos.org/plosone/s/figures#loc-tools-for-figure-preparation.

NAAS will assess whether your figures meet our technical requirements by comparing each figure against our figure specifications.

# Author comment: Author response to Decision Letter 3

**DOI:** 10.1371/journal.pone.0359078.r006

8 Sep 2026

Response to Reviewers

Manuscript ID: ONE-D-26-13257

Title: Population differences in embryonic developmental timing in the lizard Diploderma swinhonis

Dear Academic Editor and Reviewers,

We sincerely thank the Academic Editor and the reviewers for their constructive comments and valuable suggestions. We have carefully considered all of the comments and revised the manuscript accordingly. All revisions are indicated in the tracked-changes version of the manuscript, and our detailed responses to each comment are provided below.

Responses to Reviewer #3

Comment 1:

The study is interesting, and the results are significant. However, I find it hard to believe that the fact that it rains almost twice as much in Orchid as in Taiwan isn't just another variable affecting egg retention. And it’s very clear that it doesn’t directly affect the eggs, since they’re incubated under laboratory conditions, but it is affecting the females. I think the authors should make it sufficiently clear that it is the females who would be affected by the rains and the abundance of predators, which would cause them to retain their eggs. These Orchid eggs accelerate their embryonic development so that they hatch in the same number of days as they do in Taiwan.

Response to reviewer:

Thank you for this important comment. We agree that rainfall may indirectly influence egg retention and oviposition decisions through its effects on females and local nesting conditions. We have therefore added a brief discussion of previous studies showing that rainfall and soil moisture can influence egg retention and oviposition timing in lizards [45,46]. We now acknowledge that seasonal or local variation in rainfall may contribute to maternal reproductive timing and the developmental stage at oviposition.

(Lines 417–436)

Discussion

“Environmental temperature is known to influence developmental rates and reproductive traits in reptiles. However, mainland Taiwan and Orchid Island occur at similar latitudes and share broadly comparable climatic conditions and habitats, reducing the likelihood that large-scale environmental temperature differences alone explain the observed developmental divergence. Historical climatic records support this interpretation. According to the 1991–2020 climate normals provided by the Central Weather Administration (CWA)[44], Taiwan (Taichung) and Orchid Island have broadly similar annual mean temperatures (23.3°C and 23.0°C, respectively), whereas Orchid Island receives substantially greater annual precipitation than Taiwan (Taichung) (3,237.5 vs. 1,773.0 mm). Although these precipitation differences do not directly predict the pattern of prolonged egg retention observed in our study, rainfall may influence reproductive timing through its effects on female oviposition decisions and local nesting conditions. In Anolis aeneus, females can retain shelled oviducal eggs during drought, whereas rainfall facilitates oviposition when soil moisture is sufficient [45]. Similarly, García-Collazo et al. [46] found that egg retention was negatively related to precipitation, whereas environmental temperature had no significant effect. Thus, although the higher annual precipitation on Orchid Island does not readily explain its prolonged egg retention, seasonal or local variation in rainfall and soil moisture may influence maternal reproductive timing and the developmental stage at oviposition. The subsequent embryonic developmental differences observed after oviposition are therefore more likely to reflect population-specific developmental dynamics than broad-scale climatic differences.”

Comment 2:

I recommend publishing the study, and I would even suggest that the authors review the final table they present to see if it might be better to present it as a line or scatter plot (with the respective standard deviations).

Response to reviewer:

Thank you for this helpful suggestion. We carefully considered whether Fig 6 would be better presented as a line or scatter plot with standard deviations. However, we believe that the format is more appropriate for our study because Fig 6 is intended to provide the developmental data and statistical comparisons across sampling stages and populations, rather than to emphasize a continuous relationship or trend. We therefore retained Fig 6 in its current format to allow readers to directly compare the developmental stages and associated variation among populations.

Attachment Submitted filename: 1. Response to reviewers (1).docx

# Editor report: Decision Letter 3

**DOI:** 10.1371/journal.pone.0359078.r007

**Authors:** Renato Filogonio

10 Sep 2026

Population differences in embryonic developmental timing in the lizard Diploderma swinhonis

PONE-D-26-13257R3

Dear Dr. Huang,

We’re pleased to inform you that your manuscript has been judged scientifically suitable for publication and will be formally accepted for publication once it meets all outstanding technical requirements.

Within one week, you’ll receive an e-mail detailing the required amendments. When these have been addressed, you’ll receive a formal acceptance letter and your manuscript will be scheduled for publication.

An invoice will be generated when your article is formally accepted. Please note, if your institution has a publishing partnership with PLOS and your article meets the relevant criteria, all or part of your publication costs will be covered. Please make sure your user information is up-to-date by logging into Editorial Manager at Editorial Manager® and clicking the ‘Update My Information' link at the top of the page. For questions related to billing, please contact billing support.

If your institution or institutions have a press office, please notify them about your upcoming paper to help maximize its impact. If they’ll be preparing press materials, please inform our press team as soon as possible -- no later than 48 hours after receiving the formal acceptance. Your manuscript will remain under strict press embargo until 2 pm Eastern Time on the date of publication. For more information, please contact onepress@plos.org.

Kind regards,

Renato Filogonio, Ph.D.

Academic Editor

PLOS One

Additional Editor Comments (optional):

Reviewers' comments:

# Editor report: Acceptance letter

**DOI:** 10.1371/journal.pone.0359078.r008

**Authors:** Renato Filogonio

PONE-D-26-13257R3

PLOS One

Dear Dr. Huang,

I'm pleased to inform you that your manuscript has been deemed suitable for publication in PLOS One. Congratulations! Your manuscript is now being handed over to our production team.

At this stage, our production department will prepare your paper for publication. This includes ensuring the following:

* All references, tables, and figures are properly cited

* All relevant supporting information is included in the manuscript submission,

* There are no issues that prevent the paper from being properly typeset

You will receive further instructions from the production team, including instructions on how to review your proof when it is ready. Please keep in mind that we are working through a large volume of accepted articles, so please give us a few days to review your paper and let you know the next and final steps.

Lastly, if your institution or institutions have a press office, please let them know about your upcoming paper now to help maximize its impact. If they'll be preparing press materials, please inform our press team within the next 48 hours. Your manuscript will remain under strict press embargo until 2 pm Eastern Time on the date of publication. For more information, please contact onepress@plos.org.

You will receive an invoice from PLOS for your publication fee after your manuscript has reached the completed accept phase. If you receive an email requesting payment before acceptance or for any other service, this may be a phishing scheme. Learn how to identify phishing emails and protect your accounts at https://explore.plos.org/phishing.

If we can help with anything else, please email us at customercare@plos.org.

Thank you for submitting your work to PLOS One and supporting open access.

Kind regards,

PLOS One Editorial Office Staff

on behalf of

Dr. Renato Filogonio

Academic Editor

PLOS One
